# Aromatic and heterocyclic perfluoroalkyl sulfides. Methods of preparation

**DOI:** 10.3762/bjoc.6.88

**Published:** 2010-08-18

**Authors:** Vladimir N Boiko

**Affiliations:** 1 Institute of Organic Chemistry, National Academy of Sciences of Ukraine, Murmanskaya St. 5, 02094 Kiev, Ukraine

**Keywords:** halex-process, perfluoroalkylation, perfluoroalkyl sulfides, SR_F_-introduction

## Abstract

This review covers all of the common methods for the syntheses of aromatic and heterocyclic perfluoroalkyl sulfides, a class of compounds which is finding increasing application as starting materials for the preparation of agrochemicals, pharmaceutical products and, more generally, fine chemicals. A systematic approach is taken depending on the mode of incorporation of the SR_F_ groups and also on the type of reagents used.

## Review

### 1. Introduction

Perfluoroalkyl sulfides of aromatic and heterocyclic compounds have been an important aspect in the general development of organofluorine chemistry over the last twenty years.

Alkyl aryl sulfides containing partly fluorinated aliphatic moieties have been widely used for a number of years. Their methods of preparation, for example, by the reaction of thiols with fluoro-olefins or with chloropolyfluoroalkanes are well known and have been widely used. In contrast, sulfides with fully fluorinated aliphatic chains have been limited to trifluoromethylated compounds. This was due to the unique preparation (at that time) of such compounds by means of two consecutive reaction steps: the chlorination of the side chain followed by replacement of the chlorine atoms by fluorine. This procedure enabled only the preparation of CF_3_S-derivatives because it is not possible to synthesize perchloroalkylated aromatic sulfides larger than CCl_3_S. This is currently still the case. Iodoperfluoroalkanes as perfluoroalkylating agents have only emerged rather recently.

New synthetic procedures to access this class of compounds have appeared which make use of novel intermediates. Thus, single-electron oxidation or reduction enables the generation of perfluoroalkyl radicals. Two-electron reduction of perfluoroalkyl iodides generates perfluoroalkyl carbanions, which may be stabilized by organophosphorus and organosilicon ligands and even by dimethylformamide.

**Figure 1:**
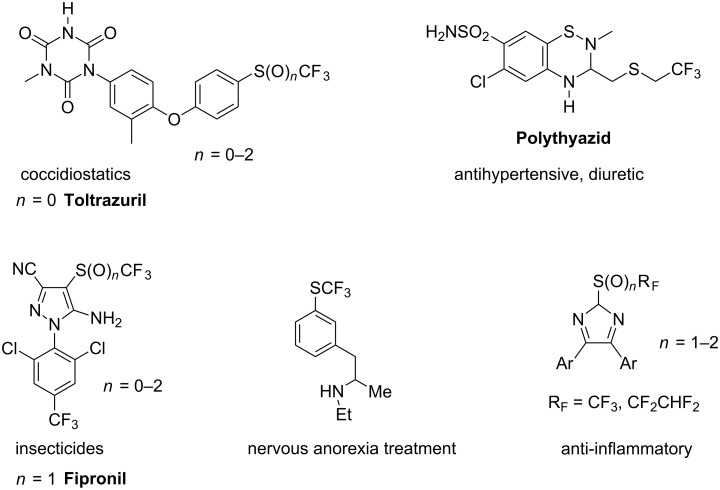
Examples of industrial fluorine-containing bio-active molecules.

**Figure 2:**
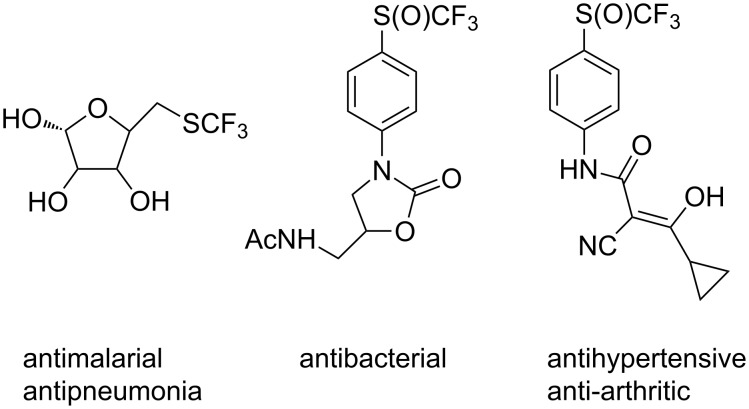
CF_3_(S)- and CF_3_(O)-containing pharmacologically active compounds.

One of the driving forces for the synthesis of perfluoroalkyl sulphides is the high lipophilic properties of perfluoroalkylthio groups (the greatest Hansch constant π = 1.44, belongs to SCF_3_ group [1]), which increases the ability of such molecules to cross lipid membranes and creates opportunities for the modification of known and new drugs. Thus this group is a useful substituent in agrochemicals and pharmaceuticals [2–4]. Examples of bioactive compounds containing SCF_3_, SOCF_3_ and SO_2_CF_3_ groups are shown in Figure 1 and Figure 2.

**Figure 3:**
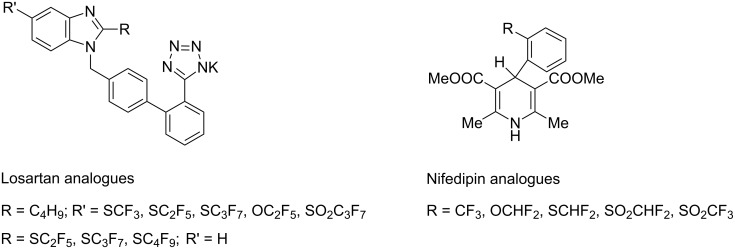
Hypotensive candidates with SR_F_ and SO_2_R_F_ groups – analogues of Losartan and Nifedipin.

The synthesis of a large number of potential hypotensive agents containing SR_F_ and SO_2_R_F_ groups of the 1,4-dihydropyridine class and also of Losartan (Dup 753) analogues which are used clinically for the treatment of cardiovascular diseases have also been developed [5–6] (Figure 3).

**Figure 4:**
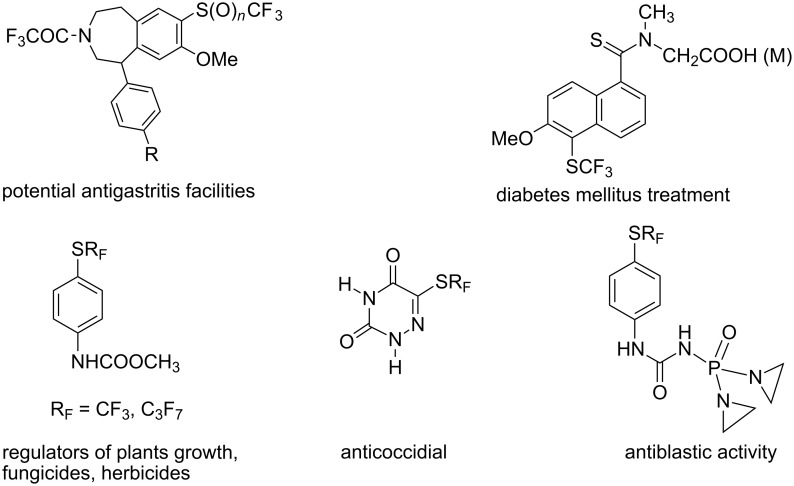
The variety of the pharmacological activity of R_F_S-substituted compounds.

**Figure 5:**
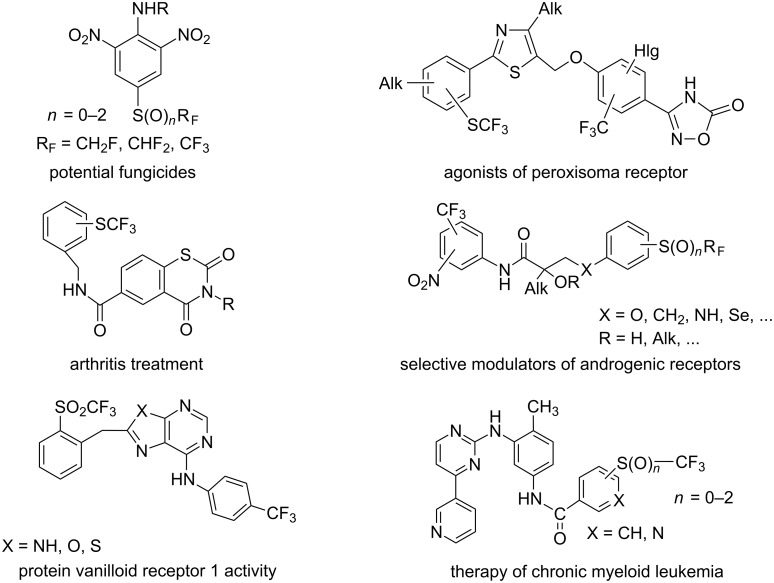
Recent examples of compounds containing R_F_S(O)*_n_*-groups [12–18].

Other patented compounds containing perfluoroalkyl thio substituents are illustrated in Figure 4 and Figure 5 along with their pharmacological functions [7–11].

These examples represent only a small number of the vast array of organic compounds with SR_F_, SOR_F_ or SO_2_R_F_ groups which display pharmacological activity and interest in such analogues continues to grow.

Previous reviews in this area are either dated [19] or focus on specialist aspects such as perfluoroalkyl radicals [20–22], fluorinated carbanions [23], organometallic compounds [24–25], perfluoroalkyl sulfenyl halides [26], perfluoroalkyl silicon reagents [27–32], the trifluoromethylthio anion [29] or electrophilic perfluoroalkylating agents [33]. Others are devoted to particular methods such as trifluoromethylation initiated by sodium dithionite [34] or the electrochemical introduction of fluoroalkyl groups in organic molecules [35]. Moreover, many of the reviews on the subject are very general [28,30,32,36].

The present work reviews synthetic methods employed to prepare aromatic and heterocyclic perfluoroalkyl sulfides and is systematized depending on the mode of constructing the SR_F_ groups and also on the nature of the starting materials.

1. The halogenation of SAlk-derivatives with subsequent replacement of the halogen atoms by fluorine.
2. The introduction of SR_F_-moieties into aromatic compounds by both electrophilic and nucleophilic reagents.
3. Various modes of perfluoroalkylation of organosulfur compounds including cationic, anionic, radical and ion-radical variants.

### 2. Substitution of halogen atoms by fluorine in aryl-α-polyhalogenoalkyl sulfides

Substitution of the halogen atoms in SAlk_Hlg_ groups (mainly chlorine) using antimony trifluoride [37], is the oldest method of perfluoroalkylsulfide preparation and is still commercially significant.

**Scheme 1:**
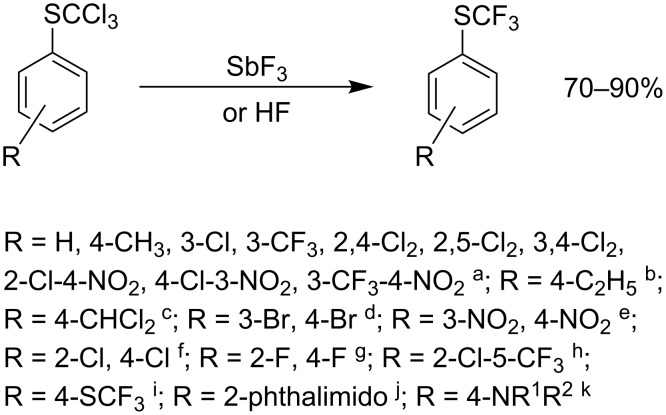
Fluorination of ArSCCl_3_ to corresponding ArSCF_3_ derivatives. For references see: ^a^[38–43]; ^b^[41–42]; ^c^[43]; ^d^[44]; ^e^[38–43,45–47]; ^f^[38–43,48–49]; ^g^[49–50]; ^h^[51]; ^i^[52]; ^j^[53]; ^k^[54].

The reaction is carried out by heating a mixture of aryl trichloromethyl sulfide with an excess of SbF_3_ in the absence of a solvent. For industrial processes, dry hydrogen fluoride is used as the fluorinating agent (Scheme 1).

The presence of halogen atoms and electron-withdrawing groups such as NO_2_, CF_3_ or COCl in the aromatic ring of trichlorothioanisole does not influence the fluorination and the reaction is not hindered by bulky ortho-substituents e.g., phthalic acid imide [53] or *N*-substituted anilines [54]. Other reactive substituents, for example 3-SCCl_3_ or 4-COCl are also fluorinated and form 1,3-bis(SCF_3_) benzene [38–40] and 4-SCF_3_-benzoic acid fluoride, respectively [55].

The use of hydrogen fluoride has some advantages. Due to its low boiling point (+19.4 °C) and good solubility in water, excess HF is easily removed from the reaction mixture. Unlike HF, reactions with SbF_3_ can be carried out in glass. The SbF_3_ must be freshly sublimed and used in a corrosion-proof vessel. Attempts to use less aggressive fluoride ion sources, e.g., KF/18-Crown-6 in CH_3_CN or KF/Bu_4_N^+^ Cl^−^ under phase-transfer conditions, have been unsuccessful [56].

**Scheme 2:**
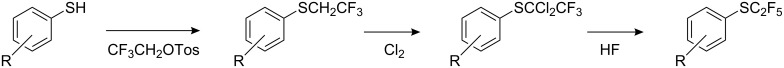
Preparation of aryl pentafluoroethyl sulfides.

The method does not give access to longer perfluoroalkyl sulfides because the required aryl perchloroalkyl sulfide precursors are not easily accessible [57–58]. However, pentafluoroethyl ethers of various thiophenols (or phenols) can be obtained by the more sequential process as shown in Scheme 2 [59].

Use of mixed (Cl/F) polyhalogenofluoro alkanes as partial fluorinated alkylating agents generates the corresponding sulfides which are appropriate precursors for subsequent conversion to perfluoroalkyl thioethers. For example, α,α-difluoro polyhalogenoalkyl sulfides and α,α-dichlorotrifluoroethyl sulfide can be obtained by reaction of thiophenols with dihalogenodifluoro methanes [60–62], per(halogenofluoro) ethanes [60,63–64] and 2,2,2-trifluorotrichloroethane.

**Scheme 3:**
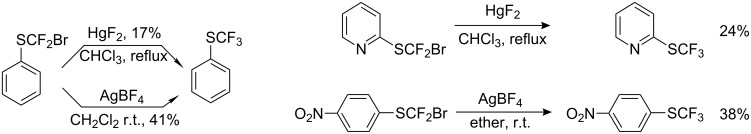
Mild fluorination of the aryl SCF_2_Br derivatives.

The Cl- and Br-substituents can then be replaced by fluorine without use of HF or SbF_3_ [61]. As shown in Scheme 3 [65], bromine to fluorine exchange is possible by the use of other heavy metal fluorides, and even by silver tetrafluoroborate under mild conditions.

**Scheme 4:**
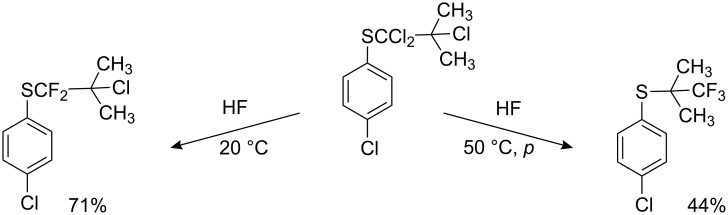
HF fluorinations of aryl α,α,β-trichloroisobutyl sulfide at various conditions.

The halex-method allows the selective preparation of α,α-difluoroalkyl aryl sulfides (and also ethers, sulfoxides and sulfones) as intermediate products in the synthesis of herbicides [66–67]. Interestingly, the reaction of anhydrous hydrogen fluoride with aryl α,α,β-trichloroisobutyl sulfide at 20 °C leads only to substitution of the α-chlorine atoms, whilst at a higher temperature and pressure a more complete fluorination with rearrangement is observed [67] (Scheme 4).

**Scheme 5:**
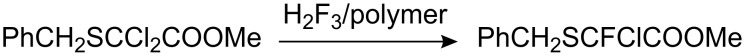
Monofluorination of α,α-dichloromethylene group.

Hydrogen fluoride/fluoride complexes such as H_2_F_3_ stabilized on a polymer [68] show even greater selectivity. For example, only one chlorine atom of the α,α-dichloromethylene group of benzyl alkyl sulfide is substituted by the reagent (Scheme 5).

Thus, halogen atoms replacement by fluorine is an effective and cheap method for preparing aromatic and heterocyclic perfluoroalkyl sulfides. Application of the appropriate conditions allows control and a degree of selectivity thus making this method an important industrial process.

### 3. Introduction of the aryl SR_F_ moiety

#### 3.1. Electrophilic introduction of SR_F_ groups

**Scheme 6:**
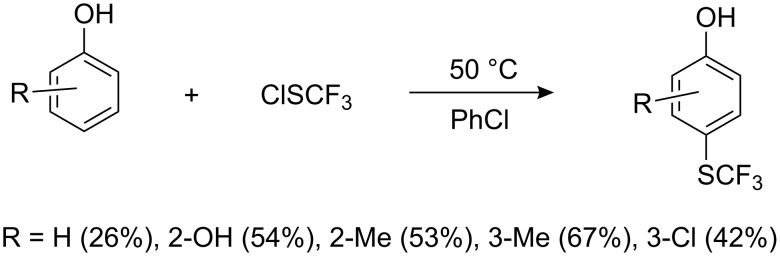
Electrophilic substitution of phenols with CF_3_SCl [69].

Perfluoroalkyl sulfenyl chlorides react with electron rich aromatic and heterocyclic compounds, to give SR_F_ derivatives. Thus, phenol, *o*-hydroquinone and their derivatives react with CF_3_SCl to yield *p*-hydroxyaryl trifluoromethyl sulfides (Scheme 6).

**Scheme 7:**
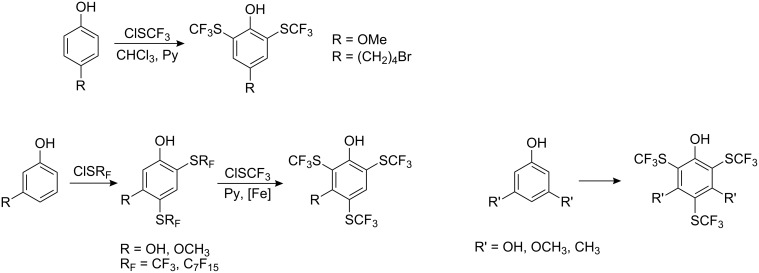
Introduction of SCF_3_ groups into activated phenols [71–74].

The best yields are achieved when electron-donating substituents are present on the ring. In the case of *m*-cresol and *m*-chlorophenol a small degree of *o*-substitution was observed. Phenol is a poor substrate in the reaction (Scheme 6) however, when FeCl_3_ was used as a catalyst the yield of *p*-HOC_6_H_4_SCF_3_ was increased, albeit only slightly (30%). A significant improvement in yield occurs (72%) when the reaction is conducted with pyridine in chloroform and at ambient temperatures (0–20 °C) [70–71]. Under these conditions and with electron-donating substituents in the phenol, two and even three perfluoroalkylthio groups can be introduced (Scheme 7).

Forcing conditions are required for the introduction of three CF_3_S-groups. This can be achieved either by activation with iron powder under pressure (or by conduction the reaction in a steel autoclave) or by the presence of two donor groups in meta-positions [71].

**Scheme 8:**
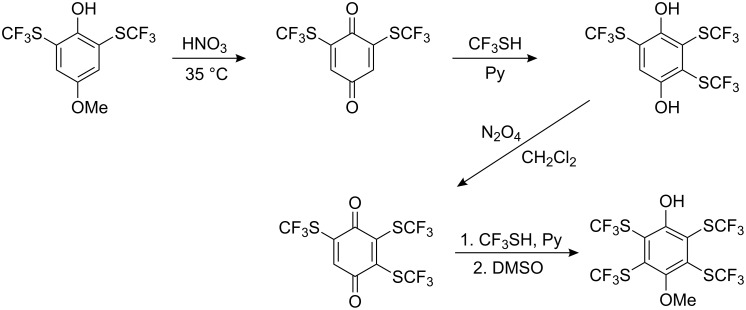
Preparation of tetrakis(SCF_3_)-4-methoxyphenol [72].

For *p*-hydroquinone, reaction with CF_3_SCl in the presence of pyridine results only in the formation of a chlorohydroquinone pyridinium species [72], and neutral conditions are required in this case [69]. For the synthesis of poly(SCF_3_) substituted *p*-hydroquinones, Scribner oxidized 2,6-bis(SCF_3_)-4-methoxyphenol to generate 2,6-bis(SCF_3_)-1,4-benzoquinone. The addition of CF_3_SH in the presence of pyridine to the bis-compound gave 2,3,5-tris(SCF_3_)hydroquinone [72] which could be subsequently converted into tetrakis(SCF_3_)-1,4-hydroquinone (Scheme 8).

**Scheme 9:**
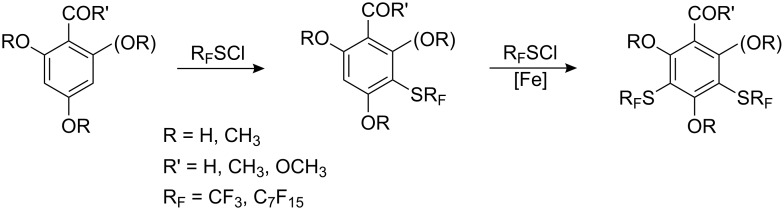
The interactions of resorcinol and phloroglucinol derivatives with R_F_SCl.

Unlike *p*-hydroquinone, resorcinols and phloroglucinols perhaps surprisingly react with R_F_SCl [75] to generate monoperfluoroalkyl thio derivatives. With iron powder as a catalyst bis(SR_F_)-derivatives can be obtained (Scheme 9).

Similarly, methyl benzoates and benzaldehydes with two and especially three hydroxyl groups form bis(CF_3_S)-substituted derivatives without of catalyst.

**Scheme 10:**
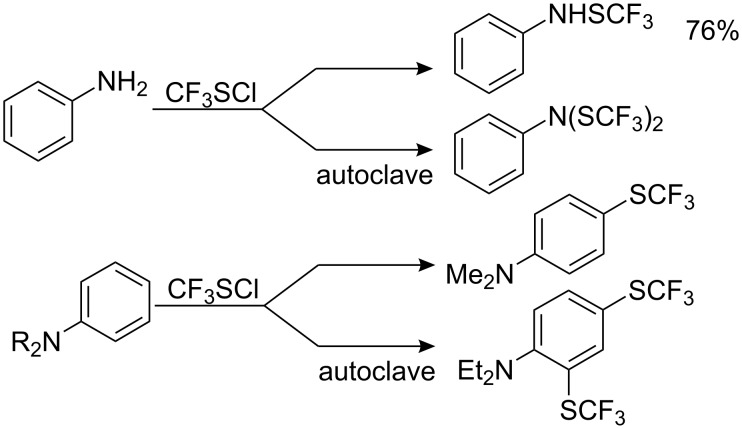
Reactions of anilines with CF_3_SCl.

Analogous reactions are observed with aniline. However, since reaction takes place in the first instance on the amino group [74,76], for the introduction of SCF_3_ group into the aromatic ring the amino function must be protected. Mono-*N*-substitution is insufficient: *N*-methyl aniline, *N*-(SCF_3_)aniline and *N*(Ac)-*m*-toluidine all yield mainly *N*-(SCF_3_)-derivatives, and only a small amount of aromatic CF_3_S-substitution is observed [74]. The best results are achieved [70,74] with *N*,*N*-bis-substituted aniline (Scheme 10).

**Scheme 11:**
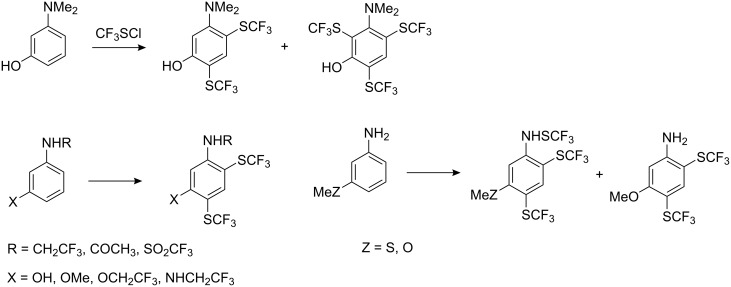
Trifluoromethylsulfanylation of anilines with electron-donating groups in the meta position [74].

The introduction of strong electron-donating meta groups significantly activates the aromatic nuclei not only for *N*,*N*-bis-substituted anilines but also for *N*-monosubstituted substrates and even those with a free NH_2_ group (Scheme 11).

In naphthalene and benzophenone derivatives only those rings containing hydroxy or amino groups undergo perfluoroalkylsulfanylation [74–75]. Other electron-donating substituents on the aromatic ring are not so activating for reaction with CF_3_SCl. For example, thiophenol [76] forms only phenyltrifluoromethyl disulfide [70]. The presence of a methyl group and halogens requires high temperatures (100–200 °C) and the presence of catalysts (HF or BF_3_) for reaction and yields of the corresponding aryltrifluoromethyl sulfides are only 25–60%. Both toluene and halobenzenes lead to mixtures of isomers [70].

**Scheme 12:**
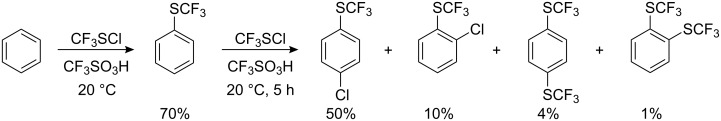
Reaction of benzene with CF_3_SCl/CF_3_SO_3_H [77].

Benzene undergoes trifluoromethylsulfanylation with trifluoromethanesulfonic acid as a catalyst even at 20 °C. However, further reaction of the resultant phenyltrifluoromethyl sulfide leads mainly to chlorination with only minor amounts of bis-(CF_3_S) products (Scheme 12).

**Scheme 13:**
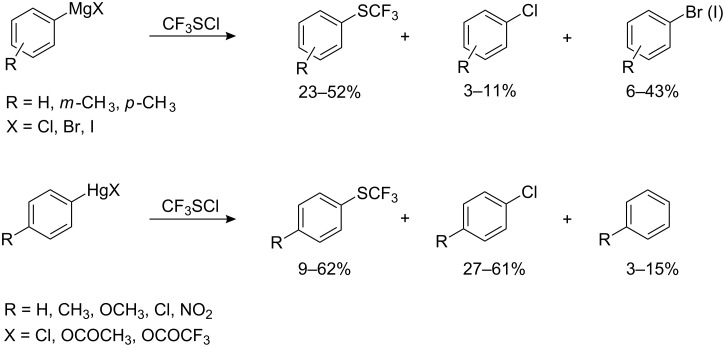
Reactions of trifluoromethyl sulfenyl chloride with aryl magnesium and -mercury substrates.

Aryl magnesium [78] and -mercury [79] compounds have been employed for the introduction of CF_3_S groups. Such reactions proceed in ether or THF at low temperatures; however, the yields of aryltrifluoromethyl sulfides do not exceed 50–60% and are accompanied with halogenated side-products (Scheme 13).

**Scheme 14:**
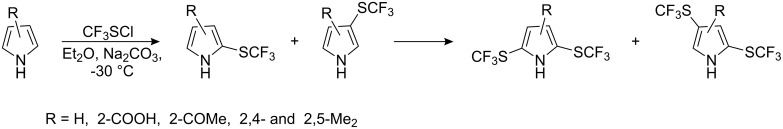
Reactions of pyrroles with CF_3_SCl.

Among heterocyclic systems, pyrroles are the best substrates for reaction with trifluoromethyl-, difluorochloro- and dichlorofluoromethyl sulfenyl chlorides. Their reactivity exceeds that of benzene and its organometallic derivatives [80]. An excess of reagent gives bis-(SCF_3_) pyrrole derivatives as shown in Scheme 14.

**Scheme 15:**
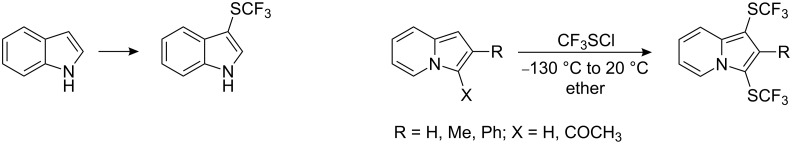
Trifluoromethylsulfanylation of indole and indolizines.

Condensed pyrroles also react readily with CF_3_SCl. Indole undergoes substitution, as expected, at the 3-position [80], while indolizine and some of its derivatives give 1,3-bis (SCF_3_)-substituted products, in some cases, in quantitative yield [81]. It is interesting to note that not only hydrogen, but also an acetyl group in the 1-position is substituted (Scheme 15).

However, no reaction occurs when there are two electron-withdrawing groups in the five-membered indolizine ring (e.g. R = Ph, and X = COPh or NO_2_). By contrast, in the case of 1-benzyl-2-methyl indolizine [81] both the pyrrole and the aromatic ring of the benzyl group undergo trifluoromethylsulfanylation. Only *N*-substitution occurs in the case of carbazole [80].

**Scheme 16:**
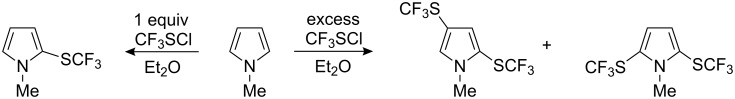
Reactions of *N*-methylpyrrole with CF_3_SCl [80,82].

*N*-Methylpyrrole can be variously substituted depending on the conditions as illustrated in Scheme 16.

Heating *N*-methylpyrrole in CHCl_3_/Py affords the 2-SCF_3_ derivative along with a small amount of 3-SCF_3_-*N*-methylpyrrole [83]. Attempted selective introduction of the second SCF_3_ group at −30 °C with C_4_F_9_SO_3_H to 2-trifluoromethylsulfanylpyrrole was unsuccessful and gave a mixture of 2,4- and 2,5-isomers [87].

**Scheme 17:**
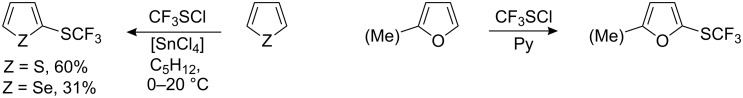
Reactions of furan, thiophene and selenophene with CF_3_SCl.

Unlike pyrroles, furan, thiophene and selenophene react with CF_3_SCl only in the presence of catalysts. For selenophene [84] and thiophenes [85] SnCl_4_ is sufficient, whilst furans require more forcing conditions usually involving prolonged heating (20 h at 60 °C) and in pyridine for activation [83–84] (Scheme 17).

**Scheme 18:**
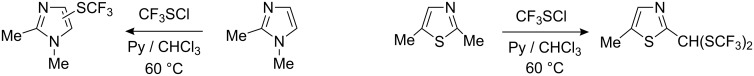
Trifluoromethylsulfanylation of imidazole and thiazole derivatives [83].

Similarly, some five membered heterocycles with two heteroatoms [*N*-Ac- and *N*-(SO_2_Alk)-thiazoles, 1-Me-2-SCH_2_CF_3_- and 1,2-Me_2_-imidazoles] undergo single trifluoromethylsulfanylation on heating (60 °C) with CF_3_SCl in a pyridine-chloroform mixture [83]. Interestingly, unlike 1,2-dimethylimidazole, the sulfanylation of 2,4-dimethylthiazole under the same conditions occurs twice on the same 2-methyl group (Scheme 18).

**Scheme 19:**
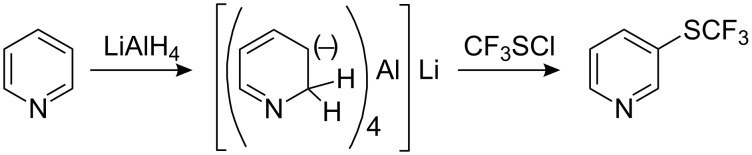
Trifluoromethylsulfanylation of pyridine requires initial hydride reduction.

Pyridine is too deactivated for trifluoromethylsulfanylation under classical conditions and to achieve substitution it is first of all necessary to convert pyridine to an anionic hydride σ-complex by reduction with LiAlH_4_ [86]. The reaction with CF_3_SCl then proceeds with difficulty [84] and mono-substituted 3-trifluoromethylsulfanyl pyridine is formed in low yield along with small amounts of the 3,5-bis(SCF_3_) derivative (~1%) (Scheme 19).

**Scheme 20:**
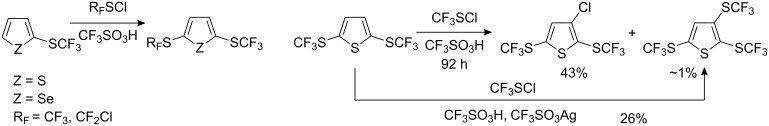
Introduction of additional R_F_S-groups into heterocyclic compounds in the presence of CF_3_SO_3_H.

**Scheme 21:**
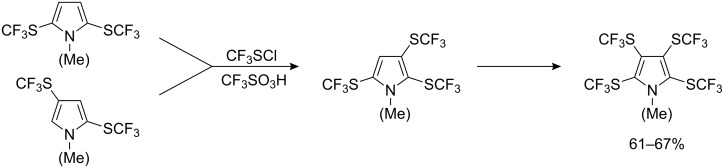
Introduction of additional R_F_S-groups into pyrroles [82,87].

Introduction of additional R_F_S-groups into heterocyclic compounds (except for pyrrole and its derivatives) occurs in the presence of perfluoroalkanesulfonic acids (Scheme 20). Incorporation of the second fluoroalkylsulfanyl group into thiophenes [85] and selenophene [84] is possible in the presence of CF_3_SO_3_H. However, reaction of CF_3_SCl with 2,5-bis(SCF_3_) thiophene in presence of CF_3_SO_3_H gives the 3-chloro-derivative as the major product. 2,3,5-Tris(SCF_3_) thiophene is accessible if CF_3_SO_3_H is added as its Ag-salt [77]. Such reactions can also be successfully carried out on pyrroles (Scheme 21).

**Scheme 22:**
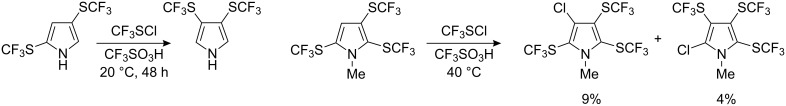
By-products in reactions of pyrroles with CF_3_SCl [82].

Prolonged reaction times lead to chlorinated products as well as products that arise from migration of the CF_3_S-groups (Scheme 22).

Thus, the reaction of perfluoroalkanesulfenyl chlorides with electron-rich aromatic and heterocyclic compounds offers an effective and comparatively straightforward method for the introduction of one or more SR_F_ groups. The reactions are more problematic however, for electron deficient substrates where competing halogenation, reduction and isomerization products often result from perfluoroalkylthiolation reactions.

#### 3.2. Nucleophilic introduction of SR_F_ groups

Anionic salts of type R_F_S^−^ M^+^ and their heavy metal complexes have been known for many years [88], however their application to the synthesis of aromatic perfluoroalkyl sulfides is comparatively recent. For example, trifluoromethylthiomercury and trifluoromethylthiosilver react with aliphatic halogenides to generate aliphatic and benzylic trifluoromethyl sulfides [89–92].

**Scheme 23:**
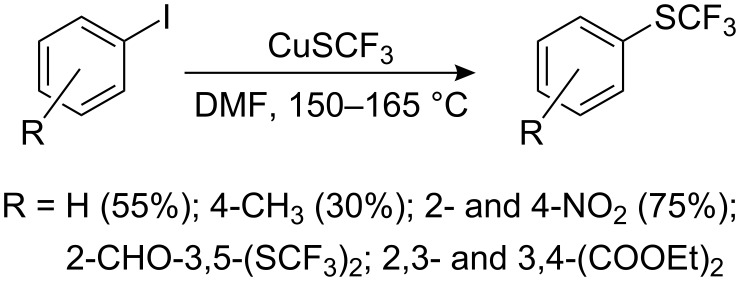
Reaction of aromatic iodides with CuSCF_3_ [93,95].

It is well known that the reaction of non-activated aryl halides with phenols, thiophenols and amines are catalyzed effectively by copper (Ullmann reaction). L. M. Yagupol’skii [93–97] developed a related protocol for trifluoromethylsulfanylation of aromatic and heterocyclic compounds using trifluoromethylthiocopper (Scheme 23).

**Scheme 24:**
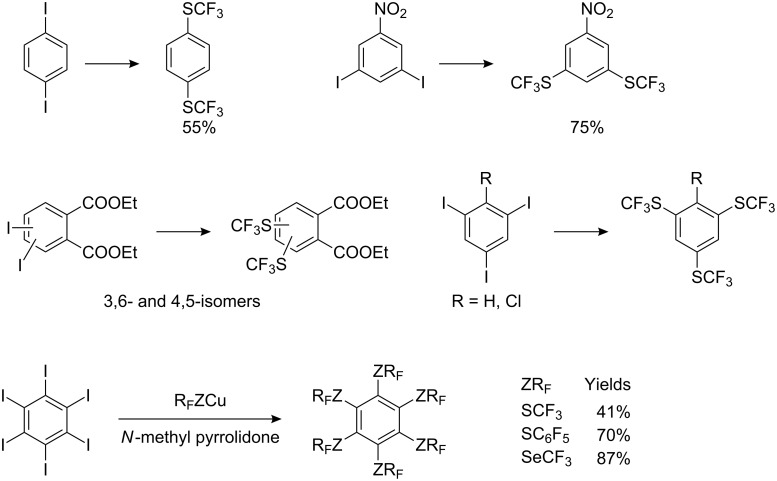
Reaction of aromatic iodides with R_F_ZCu (Z = S, Se), R_F_ = CF_3_, C_6_F_5_ [93,95–96].

The reaction is carried out by heating in a polar solvent (e.g. DMF, quinoline or *N*-methyl pyrrolidone) and the substrate can contain electron-donating or electron-withdrawing groups. Electron-withdrawing groups activate the iodo atom and consequently, give better yields (70–75%). 2-Trifluoromethylsulfanylpyridine, 6-trifluoromethylsulfanylquinoline [93] and 1-trifluoromethylsulfanylnaphthalene [97] are obtained in good yields (60–70%) by this method. Multiple aromatic iodine substituents result in multiple substitution by SCF_3_ (Scheme 24).

In the cases of triiodo derivatives, the yields generally do not exceed 30%. Thus, the synthesis of 1,3,5-tris(SCF_3_)benzene is more efficient via 3,5-bis(SCF_3_)-iodobenzene [93]. Hexaiodobenzene reacts with CuSCF_3_ to form hexakis(trifluoromethylsulfanyl)benzene in modest yield (41%). However, with CuSC_6_F_5_ and CuSeCF_3_ the corresponding hexa-substituted thio- and seleno-derivatives are obtained in yields of 70–90% [96].

**Scheme 25:**
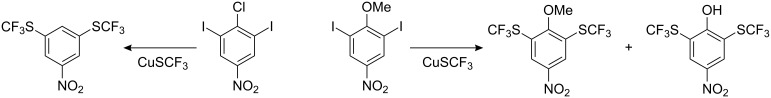
Side reactions during trifluoromethylsulfanylation of aromatic iodides with CF_3_SCu [98].

It should be noted that the interaction of CuSCF_3_ with aromatic iodides is sometimes accompanied by side-reactions. For example, the introduction of CF_3_S groups into 2,6-diiodo-4-nitrochlorobenzene and 2,6-diiodo-4-nitroanisole involve simultaneous reduction and substitution (Scheme 25).

Trifluoromethylthiocopper is obtained by reaction of CuBr with AgSCF_3_ [93,99], the latter is generated from silver fluoride and carbon disulfide [90,100].

**Scheme 26:**
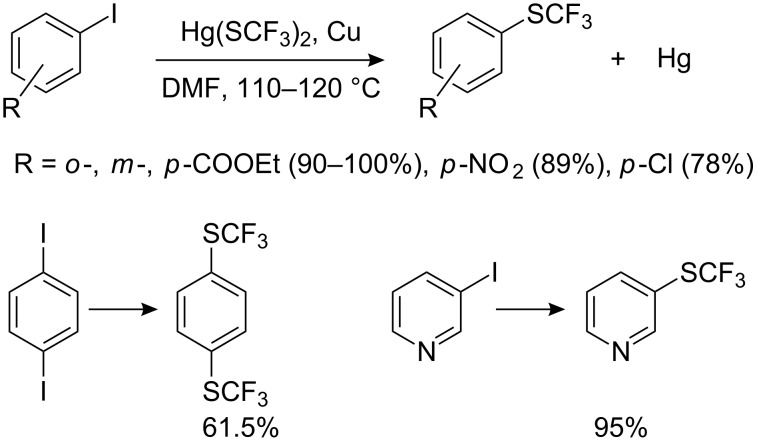
Reactions with in situ generated CuSCF_3_.

To simplify the process, Remy [101–102] suggested carrying out the synthesis of aryltrifluoromethyl sulfides by generation CuSCF_3_ (from trifluoromethylthio mercury and -copper) in situ with the aryl halides. This not only reduces the number of steps but also increases the overall efficiency (Scheme 26).

Aryl bromides can also be used but require higher temperatures (150–190 °C) and more polar solvents. Under such forcing conditions compounds containing both electron-withdrawing and electron-donating groups can now be used effectively. In the case of *p*-bromo-*N*,*N*–dimethylaniline an excess (3 equiv) of the reagent was used. Aromatic chlorides do not react under these conditions. Thus, this method allows the selective substitution of different halogens by varying the temperature.

Since the original work on trifluoromethylthiocopper and trifluoromethylthiomercury [93,95–96,101–102], other nucleophilic reagents and new methods have been developed. For example, Clark et al. have used CuSCF_3_ adsorbed onto Al_2_O_3_ [100], whilst Munavalli et al. have employed the acetonitrile adduct CF_3_SCu·CH_3_CN [103] for the reaction with *m*-iodobenzoic acid and its methyl ester [104].

**Scheme 27:**
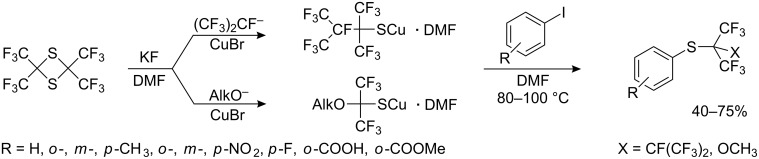
Perfluoroalkylthiolation of aryl iodides with bulky R_F_SCu [105].

Bulky perfluoroalkylthiocopper reagents, derived from 2,2,4,4-tetrakis(CF_3_)-1,3-dithietane, hexafluoropropene and alcohols in the presence of KF or CuBr, have been also used for reaction with substituted iodobenzenes (Scheme 27).

**Scheme 28:**
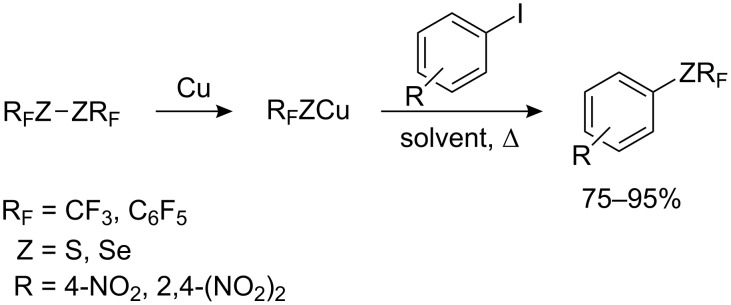
In situ formation and reaction of R_F_ZCu with aryl iodides.

A variety of perfluoroalkyl- and perfluoroarylcopper mercaptides and selenides have become more accessible, prepared by cleavage of the corresponding disulfides and diselenides with copper powder [94]. The resultant R_F_ZCu reagents complexed with DMF or *N*-methylpyrrolidone, are quite stable and can be stored without decomposition, can be used for the production of aryltrifluoromethyl-, arylpentafluorophenyl sulfides and -selenides from the corresponding iodobenzenes (Scheme 28) [94].

**Figure 6:**
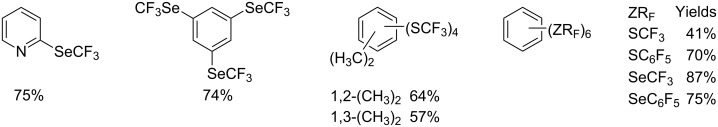
Examples of compounds obtained using in situ generated R_F_ZCu methodology [94].

The compounds shown in Figure 6 have been synthesized by this method.

**Scheme 29:**
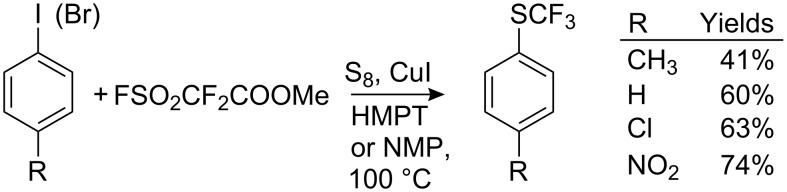
Introduction of SCF_3_ group into aromatics via difluorocarbene.

An alternative approach for the generation of CF_3_SCu involves heating of methyl fluorosulfonyl difluoroacetate in polar aprotic solvents to generate difluorocarbene, which in the presence of CuI and sulfur, forms trifluoromethylthiocopper [106]. Subsequent reaction with aryl halides results in the corresponding trifluoromethylsulfanyl derivatives (Scheme 29).

**Scheme 30:**
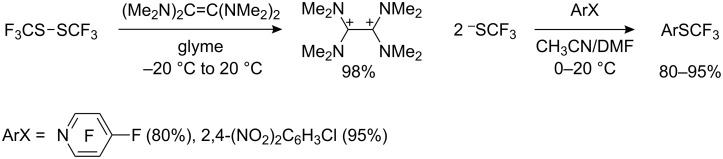
Tetrakis(dimethylamino)ethylene dication trifluoromethyl thiolate as a stable reagent for substitution of aromatic halides.

Reduction of bis(perfluoroalkyl)disulfides with tetrakis(dimethylamino)ethylene produces tetrakis(dimethylamino)ethylene dication stabilized perfluoroalkyl thiolates. In contrast to the corresponding potassium and tetramethylammonium salts [29], this compound is stable and can be isolated in a pure state [107], and reacts with activated aryl halides to form the corresponding trifluoromethyl sulfides often in quantitative yields (Scheme 30).

**Scheme 31:**
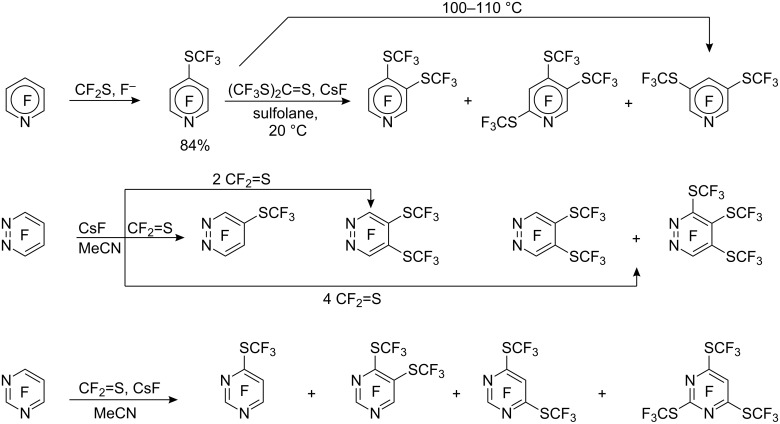
The use of CF_2_=S/CsF or (CF_3_S)_2_C=S/CsF for the introduction of CF_3_S groups into fluorinated heterocycles.

Dmowski and Haas used the reaction of thiocarbonyl difluoride with metal fluorides, to generate the trifluoromethylthiolate anion [108] for introduction into activated perfluoroheterocyclic compounds. Thus, reaction of CF_2_S/CsF with pentafluoropyridine under mild conditions gave the 4-substituted product [109]. However, for the subsequent introduction of additional SCF_3_ groups this system is not suitable due to effective self-condensation of thiocarbonyl difluoride (CF_2_=S) at higher concentrations. For this purpose the trimer of thiocarbonyl difluoride, bis(trifluoromethyl)trithiocarbonate (CF_3_S)_2_C=S, is more stable and reacts with CsF in sulfolane to generate CF_3_S^−^ anions [110]. However, the use of this reagent leads to mixtures of products (Scheme 31).

Whilst reaction of CF_2_=S/CsF (or its trimer) with tetrafluoropyridazine allows for the selective formation of mono-, di- and tri-(SCF_3_) substituted products, the analogous reaction with tetrafluoropyrimidine results in a mixture of polyfluoropyrimidine derivatives [111] (Scheme 31). Interestingly, the reaction of (CF_3_S)_2_C=S/CsF with *C*,*N*-bis(pentafluorophenyl) imidoyl chloride leads to introduction of the SCF_3_ group into the pentafluorophenyl ring along with substitution of the imidoylic chlorine atom [112].

**Scheme 32:**
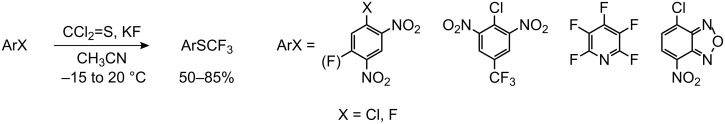
One-pot synthesis of ArSCF_3_ from ArX, CCl_2_=S and KF.

A considerable improvement of this method was developed by Clark et al. [113]: No preliminary preparation of difluorothiophosgene or its trimer is necessary, the required reagents being generated in situ (from thiophosgene and KF). The reaction with activated aromatic compounds is shown in Scheme 32.

The less reactive 2-Cl-5-NO_2_ benzonitrile forms the CF_3_S-derivative in only 49% yield after many hours reflux and 2-F-5-NO_2_ benzonitrile is a by-product despite the use of a 100% excess of thiophosgene.

The use of Me_4_NF in place of KF for the generation of the CF_3_S^−^ anion in reactions with 2,4-dinitrofluorobenzene and pentafluoropyridine increases the yields of the corresponding trifluoromethyl sulfides to 90–96% [29,114]. However, with other substrates this method can be problematic due to competing side reactions.

**Scheme 33:**
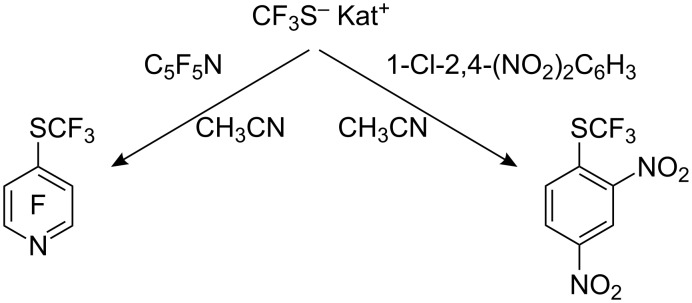
Reaction of aromatics with CF_3_S^−^ Kat^+^ [115].

A new method for the preparation of trifluoromethylthiolate anion involves the reaction of Me_3_SiCF_3_ with sulfur in the presence of a fluoride ion source [115]. The salts obtained by this method are considerably more thermally stable than those previously reported [29,110,114]. They can be treated with boiling ether or CS_2_ to remove excess sulfur and readily react at room temperature with inorganic, aliphatic and activated aromatic halides with the formation of trifluoromethyl sulfides (Scheme 33).

**Scheme 34:**
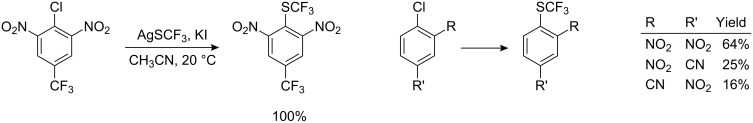
Reactions of activated aromatic chlorides with AgSCF_3_/KI.

It has already been noted that trifluoromethylthiomercury and trifluoromethylthiosilver cannot be used for the preparation of aryltrifluoromethyl sulfides, as they react only with aliphatic halides [89–92]. However, it is known [116–117], that Hg(SCF_3_)_2_ forms a complex with KI which decomposes with the formation of an unstable anion “^−^SCF_3_”. Based on this observation, Adams and Clark used a mixture of trifluoromethylthiosilver and KI (or Bu_4_NI) as a source of trifluoromethylthiolate anion for nucleophilic introduction of the trifluoromethylsulfanyl moiety into aromatic molecules [118]. Of the metal halides investigated for this reaction, the best results were obtained with KI and Bu_4_NI, whilst NaI, NaBr, and KF were ineffective. Some of these reactions are illustrated in Scheme 34.

This reagent can displace a range of activated halides, particularly bromides and iodides. For the reaction of 2,4-(NO_2_)_2_C_6_H_3_X with AgSCF_3_/KI, the reactivity of the halogens occurs in the reverse sequence: F (26%) < Cl (52%) < Br (85%) < I (97%) [118]. Presumably, coordination of the complex anionic nucleophile K^+^[Ag(SCF_3_)I]^−^ with aryl halide accelerates the reaction.

**Scheme 35:**
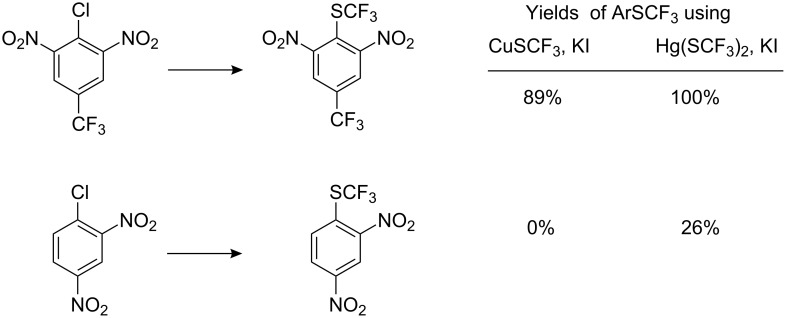
Comparative CuSCF_3_/KI and Hg(SCF_3_)_2_/KI reactions.

Trifluoromethylthiocopper and trifluoromethylthiomercury also participate in analogous reactions, CuSCF_3_ is less active than AgSCF_3_ whilst Hg(SCF_3_)_2_ displays increased reactivity as indicated in Scheme 35 [118].

**Scheme 36:**
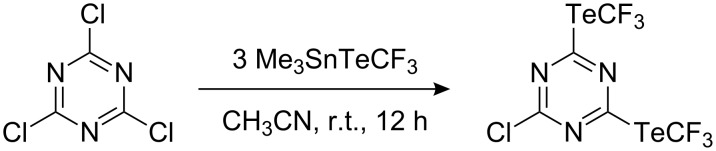
Me_3_SnTeCF_3_ – a reagent for the introduction of the TeCF_3_ group.

It should be noted that the tellurium reagent, Me_3_SnTeCF_3_, is capable of introducing the TeCF_3_ group into activated heteroaromatics [119]. In the reaction shown (Scheme 36) the use of three equivalents resulted in the introduction of only two TeCF_3_ groups.

**Scheme 37:**
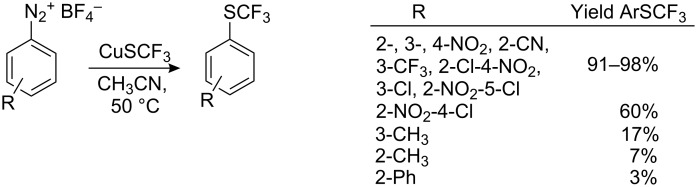
Sandmeyer reactions with CuSCF_3_.

The Sandmeyer reaction is used widely to introduce functionality into aromatic compounds. However, early attempts using trifluoromethylthiosilver as the nucleophile were not encouraging [120] with yields below 30% accompanied with deaminated side products (up to 38%). The use of trifluoromethylthiocopper was rather unsuccessful. However, with diazonium salts generated with *tert*-butyl nitrite in acetonitrile in the presence of CuSCF_3_ and BF_3_ better results were obtained [121]. Yields of the resulting aryltrifluoromethyl sulfides improved (~40–70%). The best results were observed with isolated tetrafluoroborate diazonium salts (Scheme 37), although the presence of electron-donating and bulky ortho-substituents in the aromatic ring led to reduced yields.

### 4. Perfluoroalkylation of aromatic sulfur compounds

**Scheme 38:**
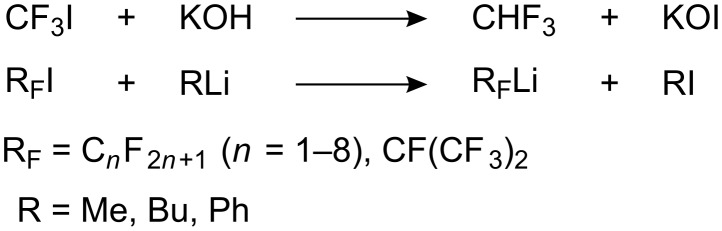
Reactions of perfluoroalkyl iodides with alkali and organolithium reagents.

Perfluoroalkyl iodides have not generally been considered as alkylating agents. Unlike R-X they show anomalous behavior in their reactions with nucleophiles. For example, the reaction of CF_3_I with alkali gives fluoroform (CHF_3_) and potassium hypoiodide (KIO) [122]. The interaction of organolithium compounds with perfluoroalkyl iodides [123–126] does not result in combination of the two alkyl species (R_F_ and R), but in trans-metallation (Scheme 38).

Such reactivity has been explained by the reverse polarization of the C–I bond in the fluorinated substrates. Because of the greater electronegativity of CF_3_ over iodine (3.3 for CF_3_ and 2.5 for the atom of iodine [127–128]), the iodine acquires a partial positive charge:


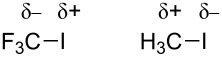


Nevertheless, Haszeldine et al., were able to carry out the perfluoroalkylations of alkylthiols. Prolonged heating of polyfluoroalkyl iodides with the sodium methylthiolate at 100–110 °C in DMSO lead to the formation of methyl polyfluoroalkyl sulfides [129]. The halophilic generated carbanion (R_F_^−^) in turn reacted with the sulfenyl iodide to generate a thioether. However, R_F_CH_3_ and R_F_H, are also obtained as by-products, which may be a result of homolytic decomposition of the perfluoroalkyl iodides at high temperature [130–131]. Similarly, reactions of R_F_I with sodium thiophenoxide (like other aromatics such as halogenated benzenes [132] or aromatic heterocycles [133]) resulted in the introduction of the perfluoroalkyl radical into aromatic rings with the formation of a mixture of isomeric R_F_-compounds.

#### 4.1. Ion-radical perfluoroalkylation

**4.1.1. Interaction of S-, Se- and Te-phenols, and diaryl disulfides with perfluoroalkyl iodides in liquid ammonia under UV irradiation**

Kornblum’s work on nucleophilic substitution in alkyl halides [134–137] and Bunnett’s reactions with non-activated aromatic substrates [138–142] (under UV irradiation) introduced the concept of the nucleophilic radical substitution mechanism (S_RN_1). The essence of this approach consists of the generation of the anionic radical RHlg^−•^, its decomposition to a radical R^•^ (Alk^•^ or Ar^•^) followed by reaction with a nucleophile.

Although perfluoroalkyl iodides have a reversed polarity, and in spite of their tendency to undergo homolytic decomposition under UV irradiation, it is probable that they are also able to react with thiolate anions by a similar mechanism. Indeed, they react readily with aliphatic, aromatic and heterocyclic thiols [143–146], and with seleno- [147] and tellurophenols [148] under UV irradiation with formation of corresponding perfluoroalkyl sulfides, -selenides and -tellurides. The original method required liquid ammonia as the solvent and Pyrex glassware. Thiophenol and its derivatives containing both, electron-donating and electron-withdrawing substituents are easily transformed to the corresponding arylperfluoroalkyl sulfides in high yields (Table 1).

**Table 1:** Interaction of thiophenols with perfluoroalkyl iodides in liquid ammonia under UV irradiation.

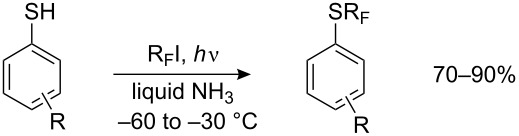			

R	R_F_	Yields of ArSR_F_, %	Ref.

H	CF_3_	76	[143]
	C_2_F_5_, *n*-C_3_F_7_, *iso*-C_3_F_7_	84, 81, 76	[144]
4-NH_2_	CF_3_	87	[146]
2-NH_2_	CF_3_	71	[143]
4-OH	CF_3_	69.5	[143]
2-OCH_3_	CF_3_	86	[98]
4-Cl	CF_3_	72	[146]
	C_2_F_5_, *n*-C_3_F_7_, *iso*-C_3_F_7_	84, 83, 65	[144]
2-SO_2_CHF_2_	CF_3_	69	[143,146]
4-SO_2_CF_3_	CF_3_	78	[143,146]
4-NO_2_	CF_3_	2.7^a^	[143,146]
		63^b^	[143,146]
2,4-Cl_2_	CF_3_	87	[149]
	C_3_F_7_	89	[149]
2-COOH	CF_3_	90	[150]
3- and 4-COOCH_3_	CF_3_, *n*-C_3_F_7_, *iso*-C_3_F_7_	70–80	[151]
3- and 4-F	CF_3_, *n*-C_3_F_7_	80–90	[152]
	*iso*-C_3_F_7_	72–75	[152]
4-NHCOCH_3_	CF_3_	96	[153]
4-NHCOOCH_3_	CF_3_, *n*-C_3_F_7_	88 (92^c^), 82 (93^c^)	[9]
	C_2_F_5_, C_4_F_9_	62, 55	[154]

^a^In a quartz flask. ^b^In a quartz ampoule at 30–45 °C. ^c^With preliminary reduction of 4,4′-bis(MeOCONH)diaryl disulfide and without the isolation of corresponding thiophenol.

α,ω-Diiodoperfluoroalkanes react at both reaction centers with the formation of bis(SAr)–derivatives containing perfluoroalkylene bridges [144,146] in yields of 80–96%.

With the exception of 4-nitrothiophenol, the reactions are independent of the type of substituents. Unlike many thiophenoxides which bear electron-withdrawing substituents (*p*-Cl, 2,4-Cl_2_, *o*-SO_2_CHF_2_ and even *p*-SO_2_CF_3_), sodium 4-nitrothiophenoxide affords 4,4′-dinitrodiphenyl disulfide under these conditions. Conversion to 4-nitrophenyl trifluoromethyl sulfide (60% yield) requires prolonged irradiation in a quartz ampoule at 30–45 °C [143]. The length of the perfluoroalkyl iodide chain has no influence, although lower yields were observed using CF_3_I in comparison with other iodoperfluoroalkanes. A branching R_F_I chain results in lower yields of the corresponding sulfides (10–15%). In the case of tertiary perfluorobutyl iodide, thiophenols are quantitatively transformed into diaryl disulfides. Such behavior of branched perfluoroalkyl iodides can be explained by the facile generation of the I^•^ radical both as a consequence of their homolytic decomposition [155] and the decomposition of in situ generated radical anions [156]: *i*-R_F_I^−•^ → *i*-R_F_^−^ + I^•^. The radical I^•^ (or I_2_) oxidizes the ArS^−^ anion to disulfide.

**Scheme 39:**
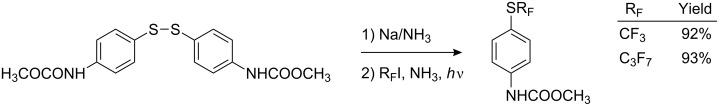
Perfluoroalkylation with preliminary breaking of the disulfide bond.

Diaryl disulfides may also be used as substrates. Although they can be trifluoromethylated directly [157], unlike dialkyl disulfides [130–131] the yields generally do not exceed 40% (except for nitro derivatives 4-NO_2_ – 58%, 2-NO_2_ – 72%). The preliminary breaking of the S–S bond can be carried out very mildly and selectively [9], without affecting other functional groups (Scheme 39).

**Scheme 40:**
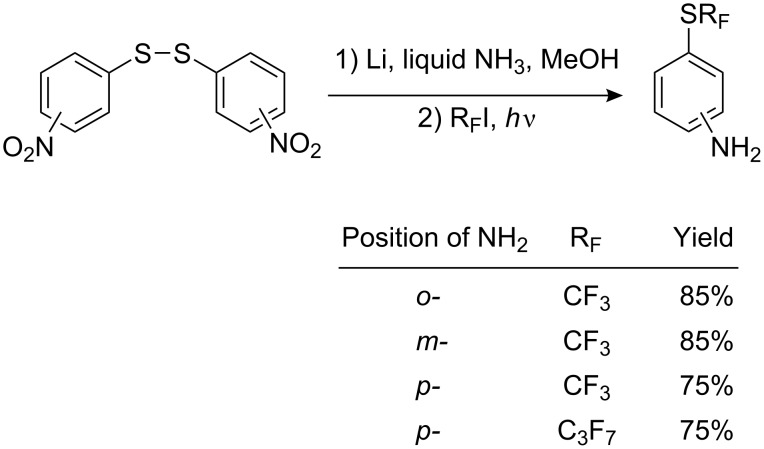
Preparation of R_F_S-substituted anilines from dinitrodiphenyl disulfides.

Perfluoroalkylthioanilines are accessible in a one-pot perfluoroalkylation reaction of dinitrodiphenyl disulfides [158–159] (Scheme 40). This method gives good yields of the desired products, higher than those from the perfluoroalkylation of amino thiophenols.

Seleno- [147] and telluro phenols [148] also react with perfluoroalkyl iodides under UV irradiation. Subsequently, it was shown that ArSeNa and ArTeNa react with perfluoroalkyl halides without irradiation to generate R_F_^•^ radicals which react with olefins [160–161]. Irradiation of polymercapto derivatives of benzene and CF_3_I in liquid ammonia gives poly(trifluoromethylsulfanyl) compounds in high yields (Table 2).

**Table 2:** UV irradiation of polymercapto benzenes with CF_3_I in liquid NH_3_.

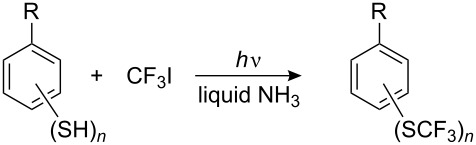			

R	Position of (SH)*_n_* and (SCF_3_)*_n_*	Yield	Ref.

Cl	2,4-	64%	[143]
COOH	3,5-	89%	[162]
CH_3_	2,4,6-	90%	[163]
NH_2_	2,4,6-	88%	[163]
OH	2,4,6-	69%	[163]

**Scheme 41:**
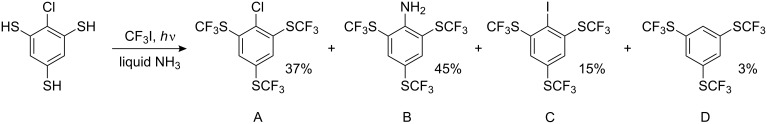
Photochemical trifluoromethylation of 2,4,6-trimercaptochlorobenzene [163].

However, the reaction of 2,4,6-trimercaptochlorobenzene with CF_3_I generates a mixture of compounds A, B, C and D as illustrated in Scheme 41. Reducing the irradiation time from 30 to 5 min does not change the product composition.

**Scheme 42:**
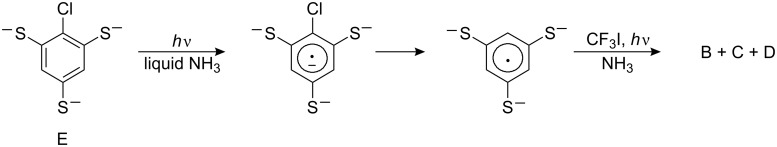
Putative process for the formation of B, C and D.

Control experiments indicate that aniline (B) is not derived from either chloro- (A) and iodo- (C)-sulfides, and iodo-product (C) is not formed from chlorosulfide (A). It is known [164] that photochemical nucleophilic aromatic substitution is promoted by electron-donating groups. Therefore, it appears most likely that the sulfides (B), (C) and (D) are produced as a consequence of loss of chloride from the intermediate radical anion as shown in Scheme 42.

Such side reactions explain the decrease of trifluoromethylation efficiency with the number of thiol groups present in a series of thiolated chlorobenzenes. The yields are 72% for 4-SH- [146], 64% for 2,4-(SH)_2_- [143] and 37% for 2,4,6-(SH)_3_- [163].

**4.1.2. Perfluoroalkylation of heterocyclic thiols**

Heterocyclic thiol form *S*-perfluoroalkyl derivatives when irradiated in liquid ammonia in the presence of iodoperfluoroalkanes. The type of heterocyclic ring and the position of the thiol group influences the reaction. More electron-deficient heterocycles require longer irradiation times (Table 3).

**Table 3:** *S*-Perfluoroalkylation of heterocyclic compounds under UV irradiation of heterocycles thiols R-Het-SH in liquid ammonia.

R	R_F_	Reaction conditions	Yield of products, %	Ref.

2-(SCF_3_)-Benzothiazole				

H	CF_3_	−60 to −33 °C, 90 min	87.5	[143]

2-(SR_F_)-Benzimidazoles				

H	CF_3_	−50 to −33 °C, 4 h	51	[165]
	C_1_–C_4_	Pyrex ampoule, 30 °C, 5 h	63–80	[154]
5-Cl	C_2_F_5_	liquid NH_3_, THF, 10 h	56	[166]

5-(SR_F_)-Benzimidazoles^a^				

2-Bu	CF_3_	liquid NH_3_, ampoule, 25–40 °C, 10 h	20–39	[154]
	C_3_F_7_			

5-(SR_F_)-6-Azauracil				

H	CF_3_	−33 °C, 45 min	77	[10]
	C_3_F_7_		76	

2-(SCF_3_)-Pyrimidines				

4,6-(CH_3_)_2_	CF_3_	−33 °C, 60 min	82	[154]
4-SH	CF_3_		61^b^	[154]
4-SH-6-CH_3_	CF_3_		58^b^	[154]
4-OH-6-CF_3_	CF_3_	Pyrex ampoule, 30–45 °C, 5 h	59	[154]
4,6-Me_2_-5-OH	CF_3_	−30 °C, 4 h,	89	[154]

^a^Received from 5-SZn salts, poorly soluble in liquid ammonia. ^b^The 2,4-bis(SCF_3_)-derivatives.

**Scheme 43:**
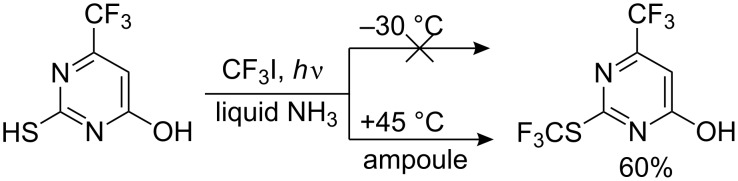
Trifluoromethylation of 2-mercapto-4-hydroxy-6-trifluoromethylyrimidine [145].

It appears that 4-hydroxypyrimidine-2-thiol does not react with CF_3_I under standard conditions. Similar to the reaction of 4-nitrothiophenol noted above [143,146], this reaction requires more forcing conditions. Other 4-hydroxypyrimidine-2-thiols behave similarly. The irradiation of an ammoniacal solution of 2-mercapto-4-oxy-6-trifluoromethylyrimidine with CF_3_I must be conducted in a Pyrex ampoule at 30–45 °C to produce the *S*-trifluoromethyl derivative (Scheme 43).

**Scheme 44:**
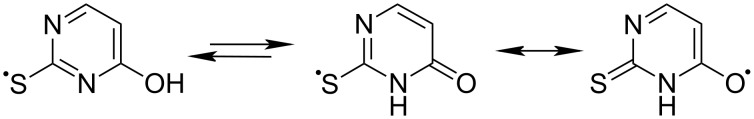
Deactivation of 2-mercapto-4-hydroxypyrimidines *S*-centered radicals.

Apparently, the reaction of these hydroxymercapto heterocyclic derivatives is complicated by stabilization of sulfur centred radicals as illustrated in Scheme 44.

In the case of 2-mercapto-5-hydroxypyrimidines, no tautomeric keto form such as that shown in Scheme 44 is possible and consequently, are perfluoroalkylated without any problems, e.g., 2-mercapto-5-hydroxy-4,6-dimethyl pyrimidine [145].

In summary, heterocyclic thiols react with perfluoroalkyl iodides with considerably more difficultly than aromatic thiols.

**4.1.3. Photochemical perfluoroalkylation in organic solvents under phase transfer conditions**

Liquid NH_3_ is a key reaction medium for the reaction of organic thiols with perfluoroalkyl iodides under UV irradiation. However, other solvents have been investigated including alcohols, acetone, acetonitrile, dioxane, THF, DMF, DMSO, HMPA and so on. Polar aprotic solvents emerge as the best. Biphasic reactions with water work well, particularly with diethyl ether and benzene (Table 4).

**Table 4:** Reaction of thiophenols RC_6_H_4_SH and mercapto heterocycles with R_F_I under UV irradiation in organic solvents and biphasic conditions.

R	R_F_	Base	Solvent	Conditions	Yields of ArSR_F_, %	Ref.

Thiophenols						

H	CF_3_	PhSNa	CH_3_OH or acetone	0–5 °C, 30 min	57.5 or 79	[143]
			CH_3_CN		89	[143]
		NaOH	CH_3_OH or acetone	0–5 °C, 30 min	43 or 49	[143]
			CH_3_CN		72	[143]
	CF(CF_3_)_2_	Et_3_N	CH_3_CN	0 °C, 30 min	88	[104]
	CF_3_	NaOH	Et_2_O/H_2_O	(Et)_3_BzN^+^Cl^−^, 20–25 °C, 30 min	54	[167]
	C_3_F_7_				78	[167]
4-Cl	C_3_F_7_	ArSNa^a^	CH_3_OH or CH_3_CN	20 °C, 30 min	61 or 81	[144]
	CF_3_	NaOH	Et_2_O/H_2_O	(Et)_3_BzN^+^Cl^−^, 20–25 °C, 30 min	61	[167]
	C_3_F_7_				85	
	*i*-C_3_F_7_				60	
	C_6_F_13_				71	
	C_3_F_7_		C_6_H_6_/H_2_O		68	[167]
4-CH_3_	CF_3_, C_3_F_7_	NaOH	Et_2_O/H_2_O	(Et)_3_BzN^+^Cl^−^, 20–25 °C, 30 min	58, 83	[167]
	C_3_F_7_		C_6_H_6_/H_2_O^b^		67	[167]
4-OCH_3_	CF_3_	NaOH	Et_2_O/H_2_O		52	[167]
4-CO_2_CH_3_	C_3_F_7_	NaOH	Et_2_O/H_2_O		71	[167]
4-NH_2_	CF_3_	NH_4_OH	NH_4_OH	−60 to 25 °C	95	[168]

2-Mercapto heterocycles^c^						

Heterocycle	R_F_	Base	Solvent	Conditions	Yield	Ref.

Benzothiazole	Cl(CF_2_)_4_	NaH	DMF	70 °C, 10 h	41.2	[169]
	Cl(CF_2_)_6_	NaH	DMF	70 °C, 10 h	61.6^d^	[169]
	C_6_F_13_	NaH	DMF	70 °C, 10 h	53.6	[169]
	C_8_F_17_				71.6	
Benzimidazole	Cl(CF_2_)_4_	NaH	DMF	70 °C, 10 h	40.6^e^	[169]
	Cl(CF_2_)_6_	NaH	DMF	70 °C, 10 h	38.2	[169]
	C_6_F_13_, C_8_F_17_	NaH	DMF	70 °C, 10 h	77.6, 78.2	[169]
Benzoxazole	Cl(CF_2_)_6_	NaH	DMF	70 °C, 10 h	15.0	[169]

^a^At ArSH + Et_2_NH or Et_3_N for 3 h, the yields are 37% and 28%, respectively. ^b^In CH_2_Cl_2_/H_2_O or CHCl_3_/H_2_O the yields are 50% and 55%, respectively. ^c^Yields of products are resulted taking into account a conversion of R_F_I. ^d^In presence of (*t*-Bu)_2_N-O^•^ the yield is 18.6%. ^e^In presence of (*t*-Bu)_2_N-O^•^ the yield is 8.6%.

Heterocyclic thiolates react more slowly with perfluoroalkyl iodides than thiophenoxides both in liquid ammonia and in organic solvents. Besides, in reactions with heterocyclic thiolates, as well as with thiophenoxides, CF_3_I is a poorer electrophile than C_3_F_7_I - even under biphasic conditions.

**4.1.4. Interaction of thiols with perfluoroalkyl bromides**

Although brominated perfluoroalkanes are cheaper and more readily available than the corresponding iodides, they react more slowly in thioether forming reactions. In general, monobrominated perfluoroalkanes do not react. However, dibromodifluoromethane, bromochlorodifluoromethane as well as 1,2-dibromotetrafluoroethane [170–171] do react with metal phenoxides and thiophenoxides via halophilic mechanisms [64], and almost always lead to mixtures of bromo and chloro containing products of mono- and di-substitution.

Their lower reactivity [88] is largely due to the greater dissociation energy of the C–Br bond (55 kcal/mol for CF_3_Br) compared to C–I (28 kcal/mol for CF_3_I) [172]. In addition, CF_3_Br has a higher reduction potential than CF_3_I and prefers to receive two rather than one electron on reduction [173].

**Scheme 45:**
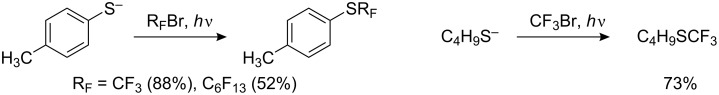
Perfluoroalkylation of thiolates with CF_3_Br under UV irradiation.

Nevertheless, it was found [174] that UV irradiation of thiolates in liquid ammonia or dimethylformamide with perfluoroalkyl bromides does result in the formation of the corresponding perfluoroalkyl sulfides as shown in Scheme 45.

Thiols with electron-donating substituents give reasonable yields, whilst *p*-chlorothiophenol produces the corresponding trifluoromethyl sulfide in low yield (~3–5%), although better yields are obtained when iodide salts are used as catalysts [175].

Wakselman et al., have shown [176] that liquid C_6_F_13_Br reacts with thiolates without any irradiation, whereas bubbling gaseous CF_3_Br through a DMF solutions of thiolates at 20 °C or heating such mixtures in an autoclave (80 °C) does not produce trifluoromethyl sulfides. Reactions between thiophenoxides and CF_3_Br are successful if carried out under pressure (CF_3_Br 2–3 atm) in DMF at 20 °C [176–178]. However, even under these conditions only thiols containing electron-donating groups in the para-position give high yields. All ethers (Table 5), even those with electron-donating groups in the ortho- and meta-positions show very poor reactivity.

**Table 5:** Yields of CF_3_Br reaction with thiophenoxides in DMF at 20 °C under pressure (2–3 atm) [178].

Substituents in thiophenols	H	4-CH_3_	4-OCH_3_	3-OCH_3_	2-OCH_3_	3-NH_2_	4-Cl	3-CF_3_	4-NHAc

Yields of ArSCF_3_, %	62	75	83	40	7	23	34	13	9

The best results arise from a combination of two factors – a pressure of CF_3_Br and UV irradiation [158,179]. Results are given in Tables 6–8. In these cases the influence of the solvent is obvious. For example, *p*-chlorothiophenol reacts poorly with CF_3_Br and 4-chloro-4′-trifluoromethylsulfanyldiphenyl sulfide is obtained as a byproduct presumably as the result of photo-substitution of chlorine in 4-trifluoromethylsulfanylchorobenzene by an S_RN_1 mechanism. HMPA suppressed this side-reaction (similar to iodobenzene with potassium diethyl phosphite [180]) and promoted trifluoromethylation (Table 6).

**Table 6:** Reactions of thiophenoxides with CF_3_Br under UV irradiation and pressure of reaction gas [179].

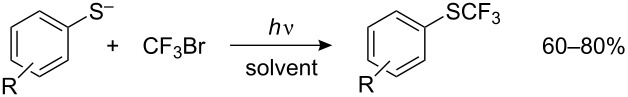							

R	Solvent	Base	*p* (atm)	*T* (°C)	Irradiation time, (h)	Conversion of ArSH, (%)	Isolated yields of ArSCF_3_ (%)

4-CH_3_	DMF	Et_3_N	4–5	10–13	1.5		82
4-NH_2_	DMF	Et_3_N	4.5–6	10–20	2		76.4
3-NH_2_	HMPA	morpholine	3–4	17–19	3.25		63.5^a^
4-NHCOMe	DMF	Et_3_N	3.5	19	2.7		69
4-NHCO_2_Me	DMF	Et_3_N	4.5–5	15–25	1.2	63	55.5
	HMPA	morpholine	2–5	8–10	2.5	73	83.6
4-Cl	CH_3_CN	Et_3_N	3–3.5	15–18	2.8	53	43^a^
	DMF	Et_3_N	3–3.5	14	1.2	100	48^a^
	HMPA	Et_3_N	4	8–10	1	100	69
	HMPA	morpholine	3–4	14–16	3.5	97	62.5
	HMPA	morpholine	3–4.5	29–30	3	36	46
	Sulfolane	morpholine	3.5	23	2	19.5	5.4
	*N*-Methyl pyrrolidone	morpholine	3.5	17	2.2	35.5	14.3

^a^Determined by GLC.

The reaction solvent is important and the yield of the trifluoromethylated product decreases in the following sequence: HMPA > DMF > CH_3_CN > *N*-methyl pyrrolidone > sulfolane [179] (Table 6). The efficiency of the combined influence of irradiation and pressure of CF_3_Br is presented in Table 7.

**Table 7:** Comparison of RC_6_H_4_SCF_3_ yields, obtained under a pressure of CF_3_Br with and without UV irradiation (DMF, *p* = 3–5 atm, *T* = 10–20 °C).

R	Irradiation time, h	Yields of RC_6_H_4_SCF_3_, %	
		
		Irradiation	Without irradiation^a^

4-CH_3_	1.5	82	75
3-NH_2_	2.2	56	23
	4	72.5	
4-NHCOCH_3_	2.7	69	9
4-Cl	1.2	48	34

^a^According to [178] (DMF, *p* = 2–3 atm, 3 h, 20 °C)

As can be seen from the data in (Table 6 and Table 7), in spite of increased product yields in general, the selectivity remains about the same. The best results are found with thiophenols, containing electron-donating substituents in the para-position. It is possible to increase the effectiveness of the *p*-chlorothiophenol reaction to ~70% by suppression of by-product formation (4-Cl-C_6_H_4_SC_6_H_4_SCF_3_-4) and by using HMPA as solvent.

Trifluoromethylation of easily oxidizable aminothiophenols can be conducted by a modified procedure. The required thiophenoxides are prepared directly prior to irradiation by reduction of the corresponding dinitrophenyl disulfides with Li/liquid NH_3_ (Table 8), in much the same way as the described above for R_F_I [158–159].

**Table 8:** Preparation of aminophenyl trifluoromethyl sulfides with CF_3_Br (3–7 atm) and UV irradiation with preliminary reduction of dinitrodiphenyl disulfides [179].

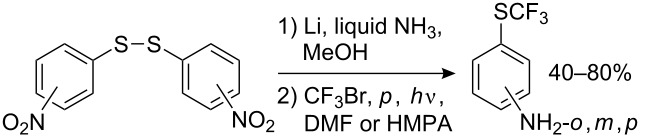					

Location of NO_2_ (NH_2_)	Solvents	*p* (atm)	*T* (°C)	Irradiation time, h	Yields of products, %

*o*-	DMF	4.6–6	10–13	7.75	40.9
*m*-	DMF	3–3.5	8–10	2.2	56^a^
	DMF	3–6	10–14	4	72.5^a^
	DMF	4–6	12–19	6.8	80.8
	HMPA	3–5	8–10	3	71.8^a^
*p*-	DMF	5–6	15–20	5	80.3

^a^Isolated as the acetyl derivative. Due to greater UV stability of CF_3_Br compared to CF_3_I, it is possible to increase the irradiation time, with a beneficial effect on the product yield.

**4.1.5. Other methods of initiating**

From the knowledge that the reaction mechanism is a single-electron transfer process involving R_F_^•^ radicals, alternative methods to photochemical initiation have been developed (see sections 4.1.1.–4.1.4.), e.g., the electrochemical reduction of perfluoroalkyl halogenides [173,181]. In the presence of thiolate anions the resulting electrophilic radicals react [182–183] to give aryl perfluoroalkyl sulfides (Table 9).

**Table 9:** Formation of aryl perfluoroalkyl sulfides by electrochemical initiated reactions of ArS^−^ with R_F_Hlg.

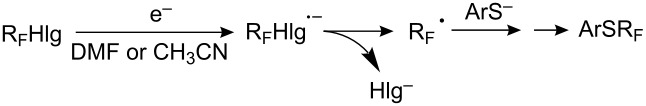				

Reagents		Yield of ArSR_F_, %		Ref.
	
ArS^−^	R_F_Hlg	On substrate	On current	

*p*-CH_3_C_6_H_4_S^−^	CF_3_I	55	300	[182]
*p*-CH_3_C_6_H_4_S^−^	C_3_F_7_I	77	270	[182]
*p*-CH_3_C_6_H_4_S^−^	CF_3_Br	40^a^	200	[182]
*p*-CH_3_C_6_H_4_S^−^	C_8_F_17_Br	63	360	[182]
*p*-ClC_6_H_4_S^−^	CF_3_I	75	250	[182]
*p*-ClC_6_H_4_S^−^	CF_3_Br	61^b^	98	[182]
*p*-ClC_6_H_4_S^−^	C_3_F_7_I	82	450	[182]
*p*-ClC_6_H_4_S^−^	CF_3_I	60	300	[181]
*p*-CH_3_OCONHC_6_H_4_S^−^	CF_3_I	33	160	[181]
Thiazole-2-S^−^	C_6_F_13_I	64^c^		[184]

^a^With a carbon-glass electrode a yield is 77%. ^b^With a carbon-glass electrode. ^c^In the presence of *p*-O_2_NC_6_H_4_CN.

The good yields for electrochemical perfluoroalkylation (especially > 100% electrochemical yield) are consistent with a radical-chain process.

Perfluoroalkyl iodides are better substrates than the bromides which give lower yields in these electrochemical reactions (Table 9). Such electrochemically initiated reactions are described in detail in a review [35].

**Scheme 46:**
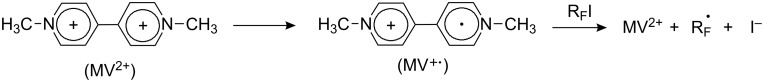
Catalytic effect of methylviologen for R_F_^•^ generation.

Another method of catalytic generation of R_F_^•^ radicals involves electron-transfer from a nucleophile to a perfluoroalkyl halide, in this case using the dimethyl dipyridinium salt (methylviologen, MV^2+^) as a catalyst. This dication is initially reduced to a radical cation, which then transfers an electron to a perfluoroalkyl iodide [185] to generate R_F_^•^ (Scheme 46). A small amount of MV^2+^ (7% relative to ArSH) is sufficient for quantitative transformation of thiols into aryl perfluoroalkyl sulfides (Table 10).

**Table 10:** Catalysis of trifluoromethylation by methylviologen [186].

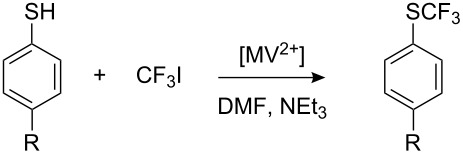			

R	MV^2+^, %	Yields of ArSCF_3_,%	
		
		With MV^2+^	Without MV^2+^

H	6.5	91.2	3
Cl	7.0	100.0	5
NO_2_	7.9	9.2	–
NHCOOMe	6.9	88.5	11

It should be noted that over-reduction of such halides will generate R_F_^−^ anions rather than the desired R_F_^•^ radicals. For example, tetrakis(dimethylamino)ethylene reacts with R_F_I to form the perfluoroalkyl anion which acts as a nucleophilic R_F_-alkylation agent for organic and inorganic substrates [187].

The use of any catalyst in the case of perfluoroalkyl iodides is of more theoretical interest, although the method can be applied in the case of poorly reactive thiophenols. In general these reactions work well (see section 4.1.6.) in common organic solvents or under biphasic conditions [188–189]. Reactions with perfluoroalkyl bromides are more sluggish. Only compounds with long perfluoroalkyl chains such as C_6_F_13_Br [178] react readily with thiolates. In the reaction of gaseous CF_3_Br with thiophenols special procedures are required (see section 4.1.4.): UV irradiation [174], pressure [178] and electrochemical stimulation [182]. Moreover, thiophenols with electron-donating substituents in the para-position give the best results. Combined pressure and irradiation [158,179] improved yields only slightly and requires special equipment. A detailed study of catalytic stimulation in reactions of bromo- and chloro-containing freons R_F_X with thiols is necessary.

**Scheme 47:**
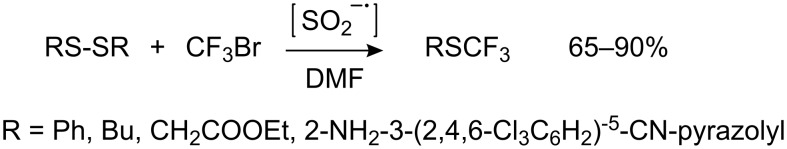
SO_2_^−•^ catalyzed trifluoromethylation.

The decreased reactivity of CF_3_Br as compared to CF_3_I can be explained, first of all, by the higher reduction potential (−2.07 V against −1.52 V for CF_3_I on a glass-carbon cathode), and secondly, by the fact that the CF_3_^•^ radical has a reduction potential (−1.80 V) close to that of CF_3_Br [173]. Thus trifluoromethyl bromide in reactions with nucleophiles or on a cathode surface accepts two electrons and is transformed to CF_3_^−^ and therefore does not react with thiolates. The SO_2_^−•^ radical anion can act as an electron mediator in such reactions. This radical anion, generated by chemical [190–193] or electrochemical [194–195] methods, causes a single-electron reduction of CF_3_Br with the formation of the necessary trifluoromethyl radical. Thus, the influence of SO_2_^−•^ sources (Na_2_S_2_O_4_, HOCH_2_SO_2_Na or SO_2_ in presence Zn and Na_2_HPO_4_ or HCOONa) on trifluoromethyl bromide in DMF in the presence of diaryl disulfides [193,196] leads to the formation of the corresponding trifluoromethyl sulfides, often in high yields (Scheme 47).

**Scheme 48:**
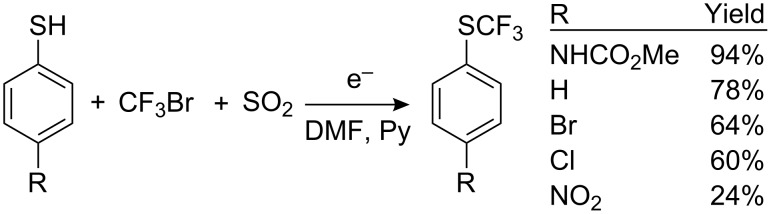
Electrochemical reduction of CF_3_Br in the presence of SO_2_ [199–200].

Related transformations with various SO_2_^−•^ sources involving R_F_I and CF_2_ClBr, CFCl_2_-CF_2_Cl in the reactions with diaryl disulfides [197] and diselenides have been reported [198]. Electrochemical studies involving the SO_2_^−•^ radical anion prove that the electron transfer to CF_3_Br takes place at a reduction potential of the mediator between −0.9 and −1.0 V which prevents the transfer of a second electron to CF_3_^•^ and the generation of CF_3_^−^ [199]. Therefore electrochemical reduction in the presence of sulfur dioxide allows the trifluoromethylation of thiophenols with the less reactive, but more readily available trifluoromethyl bromide (Scheme 48).

Although 4-nitrothiophenol is a very poor substrate (see section 4.1.1. and Table 11), it reacts with perfluoroalkyl iodides to afford 4-perfluoroalkylsulfanylnitrobenzenes in presence of NaH in DMF in almost quantitative yields [201], presumably via “hydride” catalysis.

**Scheme 49:**
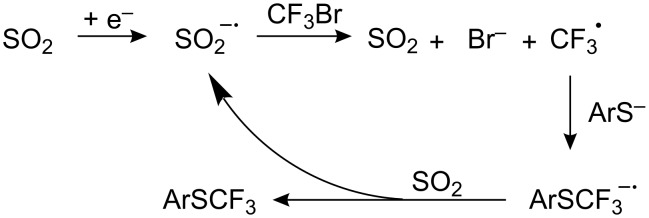
Participation of SO_2_ in the oxidation of ArSCF_3_^−•^.

The catalytic influence of SO_2_ on the reaction of ArS^−^ with CF_3_Br is not limited to the activation of the initial bromide. Sulfur dioxide can oxidize the radical anion ArSCF_3_^−•^, i.e., it can affect the rate determining step of the process [189] (Scheme 49).

This dual influence of sulfur dioxide contributes to the overall efficiency of these reactions.

**Scheme 50:**
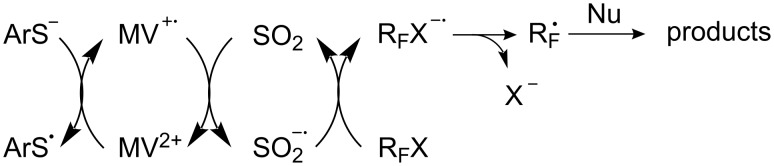
Electron transfer cascade involving SO_2_ and MV.

By comparing the possibility of two mediators (SO_2_ and MV), Koshechko et al., [202] have shown that the radical cation MV^+•^ (E_p_ = −0.4 V) easily reduces SO_2_ (E_p_= −0.9 V) to its radical anion which in turn activates CF_3_Br. Thus, a combination of both mediators generates an electron transfer cascade (Scheme 50).

Thus, bubbling CF_3_Br into a solution of thiophenol or thiocresol in DMF containing pyridine, SO_2_ and a catalytic amount of MV^2+^ 2 I^−^, results in the formation of the corresponding aryl trifluoromethyl sulfides in moderate to good yields (40–70%) [202].

Similar reactions with SO_2_, where KI or I_2_ were used instead of MV^2+^ have been carried out [202], however, the yields of PhSCF_3_ were reduced. The catalytic effect of iodide ion was discovered from UV irradiation of a reaction mixture of *p*-chlorothiophenol with CF_3_Br in different solvents [175].

The MV^2+^/SO_2_ system is effective for reactions with Freons, particularly those with C–Cl bonds such as Freon-113 (CF_2_Cl-CFCl_2_) [202].

A good example of the catalytic properties of SO_2_ has recently been shown in the reaction of 1,2-dibromotetrafluoroethane with thiophenoxides [203]. It is known that these reactions ArSCF_2_CF_2_Br and a significant amount of ArSCF_2_CF_2_H are produced. The presence of SO_2_ in the reaction promotes a S_RN_1 process which results in quantitative yields of ArSCF_2_CF_2_Br without the byproduct ArSCF_2_CF_2_H.

**4.1.6. Spontaneous perfluoroalkylation of thiols without initiators**

Since Feiring reported in 1984 that reactions of thiolate anions and perfluoroalkyl iodides can occur spontaneously without any initiator [188], the method has been extensively investigated and the reaction conditions optimized (Table 11 and Table 12). Reactions times, for example, are shortened with heating (60–70 °C) [204].

**Table 11:** Reactions of thiols RC_6_H_4_SH and HetArSH with R_F_I in organic solvents and in biphasic conditions without initiators.

R	SH, (SCat^+^)	R_F_	Base	Reaction conditions	Yields of ArSR_F_, %	Ref.

Thiophenols						

H	SNa	C_8_F_17_	—	DMF, 25 °C, 17 h	90	[188]
H	SNa	C_8_F_17_	—	DMF, 25 °C, 17 h + norbornene	77	[188]
H	SNa	C_8_F_17_	—	DMF, 25 °C, 17 h + styrene	0	[188]
H	SNa	CF(CF_3_)_2_	—	DMF, 25 °C, 17 h	76	[188]
H	SNBu_4_	C_6_F_13_	—	CH_2_Cl_2_/H_2_O, 40 °C, 4 h	48	[188]
H	SNBu_4_	C_6_F_13_	—	C_6_H_6_/H_2_O, 25 °C, 2.5 h	76^a^	[188]
H		R(CF_2_)*_n_*		DMF, conditions are not presented	56–87	[205]
4-NH_2_	SH	C_2_F_5_	K_2_CO_3_	DMF, 10 °C	84	[206]
4-F	SNa	C_10_F_21_	—	DMF, 70 °C, 1 h	97	[204]
4-F	SNa	CF_2_)_4_I	—	DMF, 25 °C,12 h, 60 °C, 1 h	86^b^	
4-Cl	SNa	(CF_2_)_8_I	—	DMF, 50 °C, 6 h		
H	SH	C_4_F_9_	NaH	DMF, 20–25 °C, 17–18 h	66	[201]
4-CH_3_	SH	C_4_F_9_	NaH	DMF, 20–25 °C, 17–18 h	77	[201]
4-OH	SH	C_4_F_9_	NaH	DMF, 20–25 °C, 17–18 h	30	[201]
4-Cl	SH	C_4_F_9_	NaH	DMF, 20–25 °C, 17–18 h	83	[201]
4-NO_2_	SH	C_4_–C_8_	NaH	DMF, 20–25 °C, 17–18 h	93–99	[201]
F_5_	SCu	CF_2_=CF	—	DMAC, 70 °C, 20 h	65	[207]
F_5_	SCu	C_8_F_17_		DMAC, 70 °C, 20 h	0	[207]
H	SeNa	CF_3_Br		EtOH, 20 °C, 2 h, olefins	2–60	[160]
H	SeNa	C_4_F_9_I–C_8_F_17_I		EtOH, 20 °C, 2 h, olefins		[160]

Heterocyclic thiols						

Heterocycle		R_F_	Base	Reaction conditions	Yields	Ref.

2-SH-benzothiazole		C_3_F_7_	NEt_3_	DMF, 55–60 °C, 3–48 h	Traces	[189]
		C_3_F_7_	NEt_3_	DMF, 20–22 °C, 120 h	59	[189]
		Cl(CF_2_)_4–6_	NaH	DMF, 70 °C, 10 h	0–4.5^c^	[169]

2-SH-benzimidazole		Cl(CF_2_)_4–6_	NaH	DMF, 70 °C, 10 h	0–3^d^	[169]

8-SNa-quinoline		C_3_F_7_	NEt_3_	DMF, 20–22 °C, 24 h	72	[189]

^a^In the presence of norbornene and styrene the yields are 30% and 0%, respectively. ^b^*α*, *ω*-Bis(SAr)perfluoroalkanes. ^c^8.5% conver. R_F_I. ^d^~3% conver. R_F_I.

Later it was found that these types of reaction can be made to proceed considerably easier and quicker (Table 12). In acetonitrile or DMF the majority of thiophenolates react rapidly with C_3_F_7_I at room temperature (from 10–15 min to 2–3 h). However, for spontaneous reaction many factors are involved such as carrying out the reaction in the dark, temperature, solvent etc. This is discussed in more detail in section 4.1.7.

**Table 12:** Reaction conditions of thiophenoxides RC_6_H_4_S^−^ Et_3_NH^+^ with R_F_I without irradiation [189].

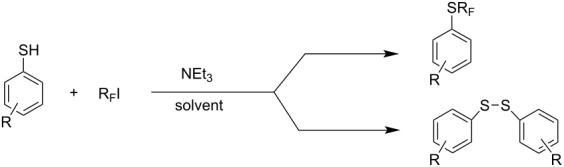								

Entry	R	R_F_	Solvent	*T* (°C)	*t* (h)	Yields (%)		
						ArSR_F_	ArS-SAr	ArSH

1	H	C_3_F_7_	DMF	19–20	2	83	3	—
2	4-NHCO_2_CH_3_	C_3_F_7_	DMF	21–22	20 min	89	3	—
3^a^	4-NHCO_2_CH_3_	C_3_F_7_	DMF	21–22	1	60	4	12
4	4-NHCO_2_CH_3_	CF_3_	DMF	21–22	1	70	9	—
5	4-NHCO_2_CH_3_	C_3_F_7_	DMF	0–5	3	17	12	30
6	4-NHCO_2_CH_3_	CF_3_	DMF	0–22	5	30	7	54
7	4-NHCO_2_CH_3_	C_3_F_7_	HMPA	0–5	3	0	12	50
8	4-NHCO_2_CH_3_	C_3_F_7_	HMPA	21–22	2	75	3	—
9	4-NHCO_2_CH_3_	C_3_F_7_	CH_3_CN	21–22	0.5	98	Traces	—
10	4-NHCO_2_CH_3_	C_3_F_7_	dioxane	21–22	2	82	2	—
11	4-NHCO_2_CH_3_	C_3_F_7_	THF	21–22	1.5	64	10	—
12	2-NH_2_	C_3_F_7_	CH_3_CN	21–30^b^	10 min	84	-	—
13	2-NH_2_	CF_3_	DMF	23–24	1	66	7	—
14	4-OCH_3_	C_3_F_7_	CH_3_CN	22–40^b^	10 min	88	6	—
15	4-Cl	C_3_F_7_	DMF	22	2	72	3	—
16	4-Cl	C_3_F_7_	CH_3_CN	21–22	3	40	12	9
17	4-COOH	C_3_F_7_	DMF	22–30^b^	10 min	72	Traces	Traces
18	4-COOCH_3_	C_3_F_7_	DMF	20	3	39	13	Traces
19	4-NO_2_^c^	C_3_F_7_	DMF	50–55	5	Traces	6	80

^a^In the dark. ^b^Spontaneous warming. ^c^Sodium thiophenoxide.

**4.1.7. Reaction mechanism**

**Scheme 51:**
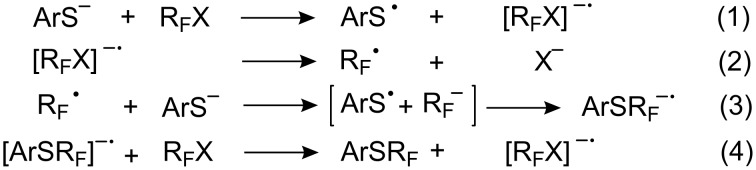
Four stages of the S_RN_1 mechanism for thiol perfluoroalkylation.

The stages of *S*-perfluoroakylation [22,35,143,188,208] can be represented as follows (Scheme 51):

The peculiar behavior of 4-nitrothiophenol [143,146] and 4-hydroxypyrimidine-2-thiol [145] unlike the more electronegative *p*-SO_2_CF_3_- and *o*-SO_2_CHF_2_-thiophenols [143] is presumably related to the ability of the nitro- and carbonyl groups to stabilize the mercapto-radicals in the radical ion pairs [^•^O_2_NArS + R_F_^−^] and [^•^O=CArS + R_F_^−^]. As a result, these radicals are less reactive, although at higher temperatures an increase in their activity is observed.

The participation of radicals is supported by the fact that the addition of nitrobenzene [178] or di-*tert*-butylnitroxide [169] inhibits the reaction. The addition of olefins such as norbornene or styrene [188] has a similar effect and perfluoroalkyl derivatives of these olefins have been identified in the reaction products. The formation of radicals in the reaction of PhSeNa with perfluoroalkyl halides (PhSe^•^ and R_F_^•^) has been firmly established from their interception by unsaturated compounds [160].

Further confirmation of a radical mechanism was obtained by studying the reaction without an initiator (Table 12 and Table 13). The decrease of reaction temperature, carrying out the reaction in the absence of light, the presence of electron-withdrawing substituents in the thiol ring and use of low-polar solvents all led to lower ArSR_F_ yields. Also replacement of C_3_F_7_I for CF_3_I leads to a slower reaction and reduced yields of aryl perfluoroalkyl sulfides. In spite of heptafluoropropyl iodide being a stronger oxidant than CF_3_I [182,209], greater amounts of diaryl disulfides are obtained only with CF_3_I. The factors listed above influence the yields of diaryl disulfides in a different way. They either do not change (in darkness), or they even slightly increase (from 3–4 to 12–13%).

These observations point towards the rate determining step of the reaction [189]. Two steps (Scheme 51), i.e., the rapid fragmentation of the radical anion R_F_X^−•^ (Equation 2) [173] and recombination of the electrophilic radical R_F_^•^ with the ArS^−^ anion (Equation 3) are fast and cannot therefore be rate limiting.

Since all experimental factors (light, temperature, solvent etc.) have an inverted influence on the yields of disulfides, it can be assumed that Equation 1, the generation of ArS^•^ is also not limiting. Therefore electron transfer from the radical anion [ArSR_F_]^−•^, Equation 4, seems to be the most likely.

**Scheme 52:**
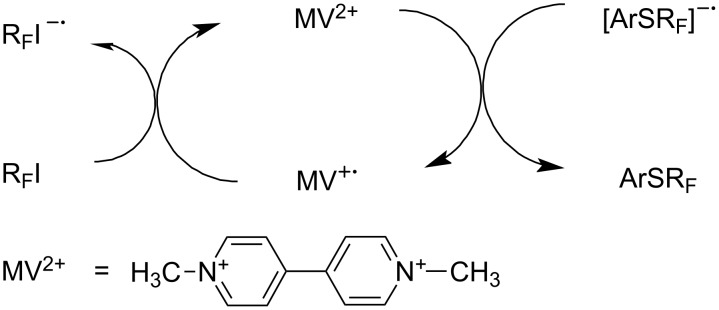
A double role of MV in the catalysis of R_F_I reactions with aryl thiols.

Homogeneous catalysis by the methyl viologen (MV) [186] supports this. This catalyst can oxidize the radical anion [ArSR_F_]^−•^ via its dication (MV^2+^) [200,202], accelerating the last step (Scheme 52).

#### 4.2. Radical perfluoroalkylation

Synthetic methods for aryl perfluoroalkyl sulfides via R_F_^•^ radicals are now described. Prolonged UV irradiation of CF_3_I solutions with diaryl disulfides in liquid ammonia results in the formation of the corresponding aryl trifluoromethyl sulfides (Table 13).

**Table 13:** UV irradiation of CF_3_I with diaryl disulfides in a sealed quartz tube [157].

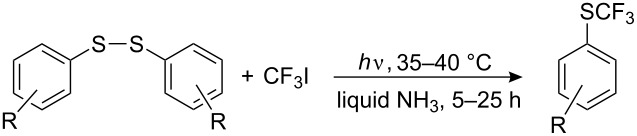		

R	*t* (h)	Yield of ArSCF_3_ (%)

H		12^a^
4-Cl	5	36.5^a^
4-NO_2_	25	58
2-NO_2_	12	72

^a^Extracted from mixtures.

**Scheme 53:**
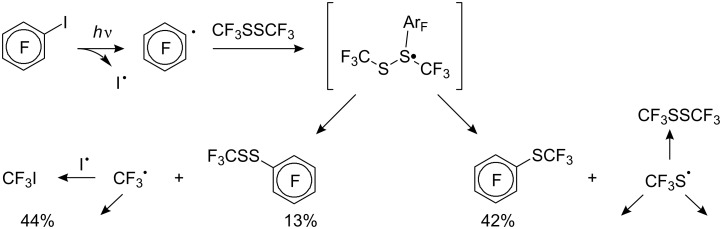
Photochemical reaction of pentafluoroiodobenzene with trifluoromethyl disulfide.

For diaryl disulfides the CF_3_^•^ radical can attack either the sulfur atom or the aromatic ring, [132,210] and thus give rise to undesired side products. Arylperfluoroalkyl sulfides are formed also in a reverse strategy from aliphatic disulfides and aryl radicals. For example, during irradiation of bis(trifluoromethyl) disulfide and pentafluoroiodobenzene [211] the product mixture contains C_6_F_5_SCF_3_, C_6_F_5_SSCF_3_, CF_3_I as well as (CF_3_S)_2_ with (CF_3_)_2_S suggesting the following reaction mechanism (Scheme 53).

**Scheme 54:**
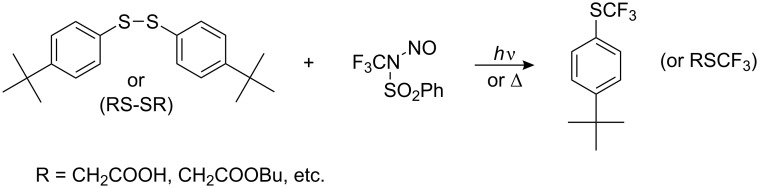
*N*- Trifluoromethyl-*N*-nitrosobenzene sulfonamide – a source of CF_3_^•^ radicals [212–213].

*N*-Trifluoromethyl-*N*-nitrosobenzene sulfonamide has been used as a source of CF_3_^•^ radicals. This reagent (obtained by reaction of CF_3_NO, NH_2_OH and benzenesulfonic acid chloride) reacts with organic disulfides under irradiation or on mild heating to give the corresponding trifluoromethyl sulfides (Scheme 54).

**Scheme 55:**
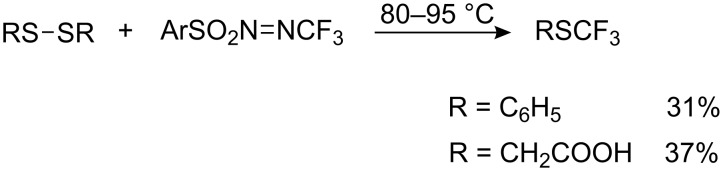
Radical trifluoromethylation of organic disulfides with ArSO_2_N=NCF_3_.

The *N*- trifluoromethylnitrososulfonamide of trifluoromethane sulfonic acid reacts similarly with aliphatic disulfides [214]. Interaction of CF_3_NO with aryl sulfonamides generates relatively stable trifluoromethyl azosulfonyl arenes ArSO_2_N=NCF_3_, which decomposed on heating to CF_3_^•^ radicals which react with organic disulfides to form trifluoromethyl sulfides [215] (Scheme 55).

**Scheme 56:**
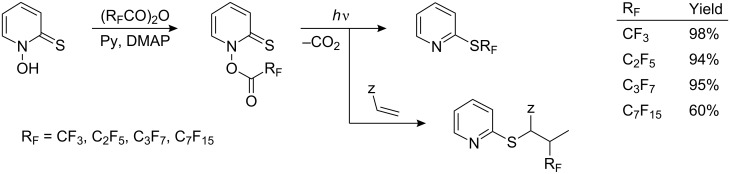
Barton’s *S*-perfluoroalkylation reactions [216].

Barton has shown [216] that the irradiation of thiohydroxamic esters of perfluorocarboxylic acids generates R_F_^•^ radicals which in the presence of olefins give addition products. However, in the absence of radical traps they attack the sulfur to yield, for example, *S*-perfluoroalkyl derivatives of pyridine (Scheme 56).

**Scheme 57:**
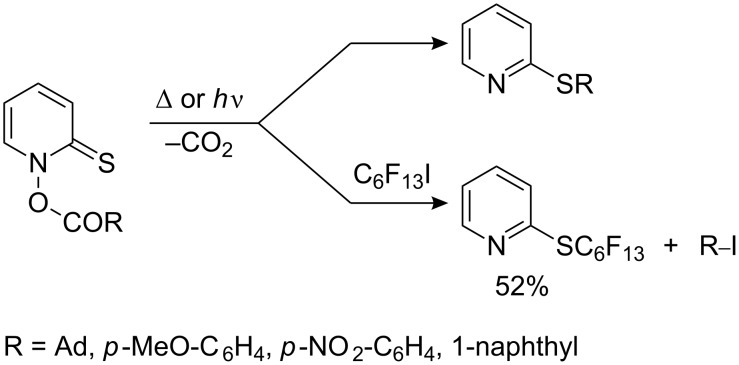
Decarboxylation of thiohydroxamic esters in the presence of C_6_F_13_I.

Decarboxylation of non-fluorinated carboxylic acid esters proceeds in a similar manner to afford 2-pyridyl sulfides. However, in the presence of C_6_F_13_I the reaction follows a different course where the perfluorinated radical attacks sulfur with the formation of the fluorinated sulfide [217] (Scheme 57).

**Scheme 58:**
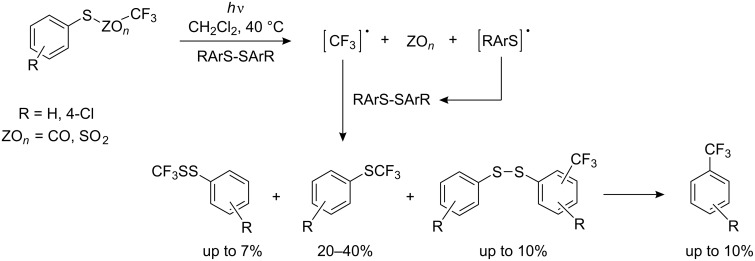
Reactions of thioesters of trifluoroacetic and trifluoromethanesulfonic acids in the presence of aromatic disulfides [218].

The irradiation of thioesters of trifluoroacetic and trifluoromethanesulfonic acids in refluxing methylene chloride results in their decarbonylation (or desulfonation in the case of CF_3_SO_2_SR) with the production of CF_3_^•^ radicals, which then react with diaryl- or dialkyl disulfides (Scheme 58).

The formation of aryl trifluoromethyl sulfides from thioesters of trifluoroacetic acid occurs in rather better yields (30–40%) than from the corresponding esters of trifluoromethanesulfonic acid (20–30%). Alkyl thioesters of trifluoroacetic and trifluoromethanesulfonic acids form AlkSCF_3_ in higher yields (up to 80%). As shown in Scheme 58, the CF_3_^•^ radical can attack at several sites. Phenyl selenide esters of trifluoromethanesulfonic acid react analogously [218].

The photochemical decomposition of trifluoromethanesulfonic and carboxylic thioesters affords CF_3_^•^ radicals which can be used to prepare trifluoromethyl sulfides [219].

**Scheme 59:**
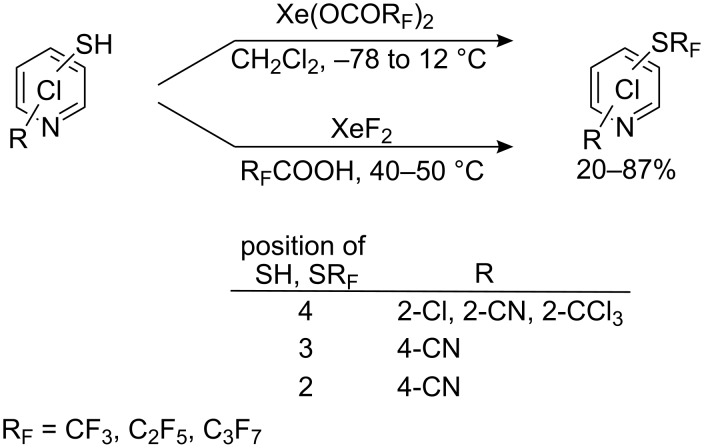
Perfluoroalkylation of polychloropyridine thiols with xenon perfluorocarboxylates or XeF_2_ [222–223].

Xenon difluoride has been used to initiate oxidative decarboxylation of perfluorocarboxylic acids for R_F_^•^ generation and with aromatic and heterocyclic compounds the perfluoroalkyl groups can also become incorporated into the aromatic ring [220]. Nevertheless, Sipyagin et al., have employed this method for the perfluoroalkylation of thiols such as polychloropyridine thiols [221]. Two different methods were used: the action of preformed xenon carboxylates (method A) or treatment of a pyridinethiol solution in R_F_COOH directly with xenon difluoride (method B). A range of isomeric perfluoroalkyl sulfides was obtained (Scheme 59).

**Scheme 60:**
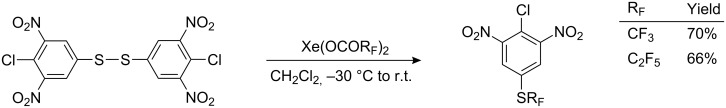
Interaction of Xe(OCOR_F_)_2_ with nitroaryl disulfide [227].

Similar reactions have been carried out with tetrafluoropyridine 4-thiol [224] and its corresponding disulfide [225–226] (40–50% yield). The formation of *S*-perfluoroalkyl derivatives with performed xenon carboxylates from nitro aromatic disulfides was also successful (Scheme 60).

Perfluoroalkylsulfinic acids can also be used for oxidative decomposition. For example, careful treatment of sodium trifluoromethylsulfinate with *tert*-butyl hydroperoxide in the presence of an organic disulfide gives the corresponding trifluoromethyl sulfide [228–229]. Aliphatic disulfides react well to give AlkSCF_3_ but problems arise with aromatic disulfides due to attack of the CF_3_^•^ radical on the aromatic rings. For example, diphenyl disulfide is converted only in 13% yield. The S/C ratio reflecting the amount of trifluoromethylation on sulfur and on the aryl ring depends on the solvent. In CH_3_CN it is 36:64, while in aqueous CH_3_CN it is 60:40. Dichlorodiphenyl disulfide gives the best ratio in favor of the sulfide in aqueous acetonitrile [228].

**Scheme 61:**
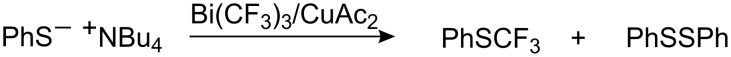
Bi(CF_3_)_3_/Cu(OCOCH_3_)_2_ trifluoromethylation of thiophenolate [230].

One final method of CF_3_^•^ radical generation involves the interaction of Bi(CF_3_)_3_/Cu(OCOCH_3_)_2_ with thiophenolate (Scheme 61).

The above methods for the synthesis of aryl perfluoroalkyl sulfides all generate electrophilic R_F_^•^ radicals which prefers to react at nucleophilic reaction centers such as S^−^, C=S or S^•^. In the case of diaryl disulfides [228] the regioselectivity of attack is less controlled due to ring delocalization.

#### 4.3. Anionic perfluoroalkylation

This method of perfluoroalkylation involves the reaction of aromatic or heterocyclic sulfur compounds with perfluoroalkyl anions, stabilized by suitable ligands, or with a reagent that generates such an anion.

Perfluoroalkyl anions are extremely unstable. For example, the CF_3_ anion decomposes at −100 °C with the elimination of F^−^ and formation of difluorocarbene, which reacts further or dimerizes [123]. Nevertheless, in the last two decades nucleophilic perfluoroalkylation of organic compounds has expanded. The problem of R_F_-lithium anion stability in synthesis has been reviewed [24]. Trifluoromethylated reagents of heavy metals and their application in organic synthesis were considered by Barton [25], whilst perfluoroalkylated [31–32] and trifluoromethylated [27–28,30] organosilicon compounds have attracted considerable interest. However, despite the large body of literature involving the use of such reagents, the synthesis of aryl perfluoroalkyl sulfides is restricted to anionic attack on sulfenyl chlorides and thiocyanates.

Various methods for the synthesis of aryl perfluoroalkyl sulfides, depending on the mode of generation of the perfluoroalkyl anion, are described below.

**4.3.1. “R_F_^−^” from a perfluorinated olefins**

**Scheme 62:**
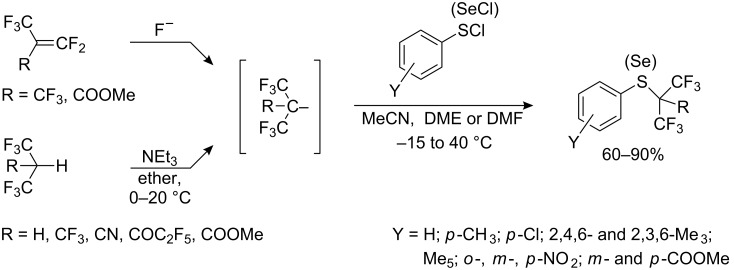
Reaction of fluorinated carbanions with aryl sulfenyl chlorides.

Relatively stable tertiary perfluoroalkyl carbanions can be prepared by addition of fluoride ion to fluoroolefins [151,231–234] or by the deprotonation of monohydroperfluoroalkanes or their derivatives [235–236] as shown in Scheme 62. Most processes involve generating the hexafluoroisopropyl carbanions with a third stabilizing group such as CF_3_ [151,231–232,236], C_3_F_7_ [233–234], as well as CN, COC_2_F_5_, COOMe [232,236]. Reactions of the resulting salts with aryl sulfenyl (or aryl selenyl) chlorides yield perfluoro- or polyfluoroalkyl sulfides (selenides).

The [C_3_F_7_(CF_3_)_2_C]^−^ anion, obtained from isomeric dimers of perfluoropropylene in the presence of KF or CsF, reacts with sulfenyl chlorides and selenyl chlorides to afford the corresponding sulfides and selenides bearing a tertiary perfluorohexyl group [233].

In the reaction of R_F_^−^ carbanions with sulfenyl chlorides high yields of sulfides are obtained when either electron-withdrawing or electron-donating substituents are present on the aryl ring. The yields of isomeric nitrophenyl perfluoro-*tert*-butyl sulfides decrease, the closer the nitro group is to the sulfur atom: *p*-NO_2_ – 86%, *m*-NO_2_ – 78% [231] and *o*-NO_2_ – 68% [232]. Both secondary and tertiary anions react [236] but nature of the counter ion is important. Thus, cesium or potassium perfluoro *tert*-butyl alkyls obtained by the addition of CsF or KF to perfluoroisobutene, give high yields of ArSC(CF_3_)_3_ [151,231–232], while the same anion, generated by deprotonation of nonafluoroisobutane (CF_3_)_3_CH with NEt_3_ gives PhSC(CF_3_)_3_ in low yield ~20% [236].

**Scheme 63:**
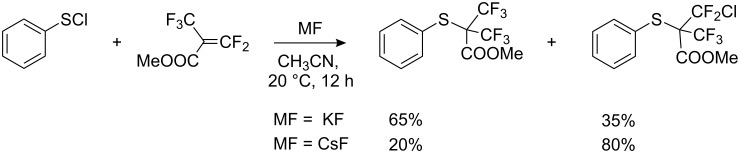
Reaction of methyl perfluoromethacrylate with PhSCl in the presence of fluoride.

In the reaction of methyl perfluoromethacrylate with PhSCl in the presence of fluoride ion, prolonged stirring gave two sulfides as shown in Scheme 63, illustrating the competition between halides (F^−^ and Cl^−^) for fluoroolefin addition [232].

**4.3.2. “R_F_^−^” from perfluoroalkyl halogenides**

**Scheme 64:**
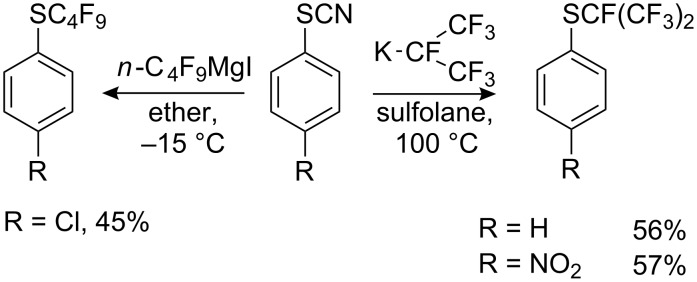
Reactions of ArSCN with potassium and magnesium perfluorocarbanions [237].

In a similar manner to alkylhalides, perfluorinated alkylhalides also form organometallic derivatives which can be used for the synthesis of perfluoroalkyl sulfides. The effectiveness of such reagents depends largely on the counterion which is illustrated below for reactions with organic thiocyanates (Scheme 64). Potassium perfluoroisopropyl (generated from CF_2_=CFCF_3_ and KF) reacts with phenyl- and *p*-nitrophenyl thiocyanates in sulfolane at 100 °C, whilst the Grignard reagent (*n*-C_4_F_9_MgI) reacts at subzero temperatures.

Cuprates react with benzyl thiocyanate but require more forcing conditions, i.e., 100 °C [237], whereas in situ generated zinc reagents R_F_ZnX react with thiocyanates at 20 °C in pyridine [238].

**Scheme 65:**
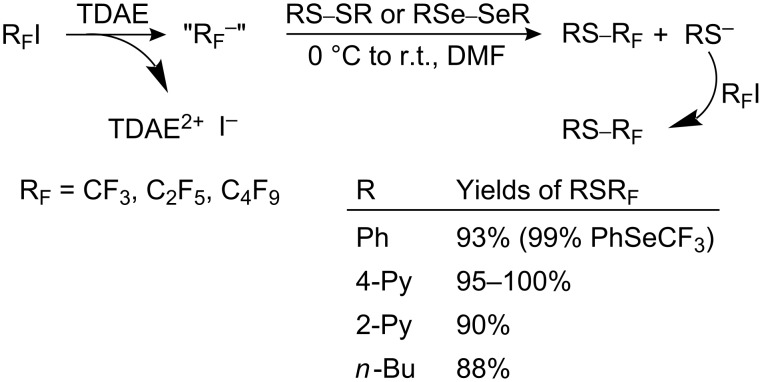
Reactions of R_F_I with TDAE and organic disulfides [239–240].

Recently, it has been shown that tetrakis(dimethylamino)ethylene (TDAE) can undergo a two-electron transfer to perfluoroalkyliodides to generate R_F_^−^ anions [187] which react with organic disulfides to afford perfluoroalkyl sulfides in high yields [239–240]. The economy of this method, as distinct from previous methods [196,241–248], lies in the fact that the thiolate released by the first nucleophilic attack on the disulfide reacts directly with a second equivalent of perfluoroalkyliodide, to form a second equivalent of the desired perfluoroalkyl sulfide (Scheme 65). This approach thus combines two principles of trifluoromethylation, i.e., nucleophilic attack of the R_F_-anion on the disulfide and reaction of a radical anion with a thiol as noted in section 4.1.

**4.3.3. “R_F_^−^” from perfluorocarboxylic acids**

A simple method for the generation of metal derivatives of perfluoroalkyl carbanions by the decarboxylation of alkali salts of perfluorocarboxylic acids, has also been used. For example, heating potassium perfluoroalkyl carboxylates in the presence of diaryl disulfides in DMF or sulfolane leads to the formation of the corresponding aryl perfluoroalkyl sulfides as summarized in Table 14.

**Table 14:** Perfluoroalkylation of aryl disulfides by decarboxylation of perfluorocarboxylates.

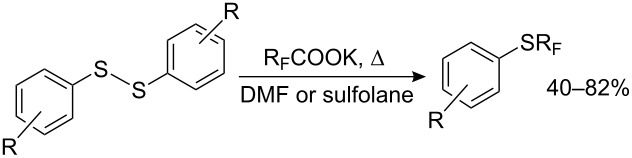					

R	R_F_	Solvent	*T* (°C)	Yield of ArSR_F_ %	Ref.

H	CF_3_	DMF	140	84	[245]
H	CF_3_	sulfolane	180–230	56	[242]
4-Me	CF_3_	sulfolane	180–230	51	[242]
4-Cl	CF_3_	sulfolane	180–230	56	[242]
4-F	CF_3_	sulfolane	180–230	82	[242]
2-Br	CF_3_	sulfolane	180–230	48	[242]
4-OMe	CF_3_	sulfolane	180–230	50	[242]
H	C_2_F_5_	DMF	145	70	[245]
4-Me	C_2_F_5_	DMF	145	50	[245]
4-NO_2_	C_2_F_5_	DMF	145	42	[245]

**Scheme 66:**
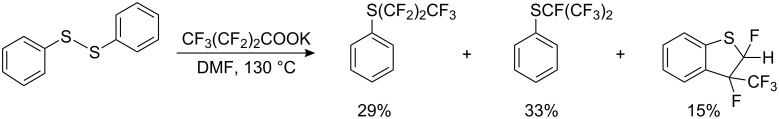
Decarboxylation of perfluorocarboxylates in the presence of disulfides [245].

Disulfides of pyridine [242], pyrimidine and naphthalene [249] have also been used in such reactions. The use of this method for longer perfluorocarboxylic acids leads to product mixtures that result from chain isomerism and cyclisation [250–251] (Scheme 66).

Polyhalogenated carboxylic acids containing fluorine together with other halogens can also alkylate disulfides. However, the results strongly depend on the structure of halogenated alkyl group. The method is successful for potassium trichloroacetate but not for difluorochloroacetate. In the latter case the corresponding sulfide PhSCF_2_Cl was found but only in trace amounts whilst PhSCCl_3_ is obtained in 80% yield [245]. The mixed haloalkyl anions appear to be less stable.

The stability and reactivity of perfluoroalkyl anions largely depend on the solvents used. For example, CF_3_MgI [252–254] and CF_3_Li [123,255–258] in diethyl ether are unstable even at low temperatures, but in coordinating solvents such as sulfolane, *N*-methylpyrrolidone, HMPA and especially, in DMF, the CF_3_^−^ anion does not decompose so readily and can be used as a nucleophilic reagent [259].

**4.3.4. “CF_3_^−^” from trifluoromethane (fluoroform)**

Trifluoromethane (fluoroform) has been used as a source of the trifluoromethyl anion. Trifluoromethane is a waste product of Teflon manufacture and it is of interest as a raw material for organofluorine chemistry [260]. However, its application has been restricted by the low stability of the CF_3_^−^ anion [123,252–255].

**Scheme 67:**
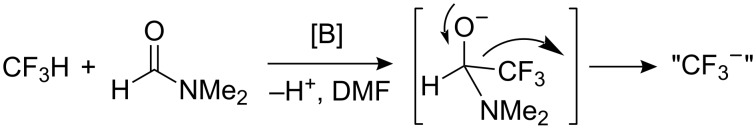
Organization of a stable form of “CF_3_^−^” anion in the DMF.

The CF_3_^−^ anion has greater stability when the counter ion is a bulky ammonium ion, and in the presence of pyrrolidone it reacts with aldehydes and ketones [261]. This suggests that an intermediate gem-aminoalcoholate is involved. The method is improved with DMF, which is also thought to form a stable aminoalcoholate intermediate (Scheme 67) [243,262–263].

This mechanism is supported by the observation that equivalent reactions do not occur in THF or DMSO [263]. Furthermore, the intermediate CF_3_ aminoalcoholate has been trapped in its protonated form and as hydrated trifluoroacetaldehyde by the action of acids, as well as trapped as a silyl ether [243]. The deprotonation of fluoroform has been applied successfully for the synthesis of aromatic trifluoromethyl sulfides and selenides, as summarized in Table 15.

**Table 15:** Reaction of the CF_3_^−^ anion derived from fluoroform with S-derivatives of thiophenols.

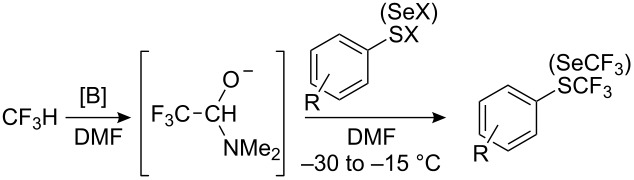				

B	R	X	Yield, %	Ref.

*t*-BuOK	H	SPh	80	[243]
*t*-BuOK	H	SO_2_Ph	90	[243]
*t*-BuOK	4-NO_2_	Cl	60	[243]
LiN(TMS)_2_/NH(TMS)_2_	H	SPh	4	[244]
N(TMS)_3_/Me_4_NF	H	SPh	6	[244]
*t*-BuOK	H	SPh	82	[244]
N(TMS)_3_/Me_4_NF	H	SePh	61^a^	[244]
*t*-BuOK	H	SePh	77^a^	[244]

^a^PhSeCF_3_.

**Scheme 68:**
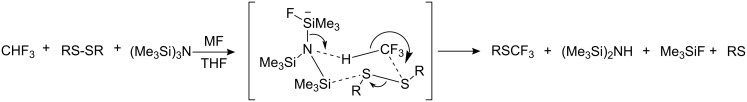
Silylated amines in the presence of fluoride can deprotonate fluoroform for reaction with disulfides [244].

Langlois et al. have used silylated amines in the presence of fluoride ion to promote fluoroform deprotonation [244]. For example, with (Me_3_Si)_3_N such reactions were possible in both DMF and THF. In the latter case stabilization of the CF_3_^−^ anion and its reaction with disulfide probably involves a transition state complex such as that depicted in Scheme 68.

**Figure 7:**
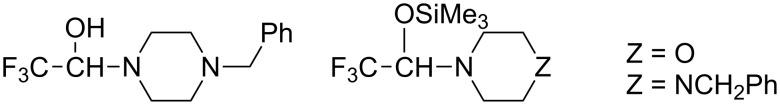
Other examples of aminomethanols [264].

In the case of trifluoromethylation of aliphatic disulfides, silazanes are the preferred reagents. However, in the case of diaryl disulfides, e.g., diphenyl disulfide, the significant formation of byproducts occurs and, PhSN(TMS)_2_ (46%) and PhSCHF_2_ (23%) are main reaction products. Other CF_3_ aminomethanols have been synthesized by Langlois et al. [264] (Figure 7).

Trifluoromethylation of disulfides by the first of them was efficient, for example, 87% in the case of PhSCF_3_ but less efficient for diselenides (PhSeCF_3_ 45%) [246]. The reaction failed with bis(4-chlorophenyl) disulfide and dioctyl disulfide where only by-products were generated.

Silylated hemiaminals are more suitable for CF_3_^−^ transfer (Table 16), although high reaction temperatures (60–80 °C) are required.

**Table 16:** Reactions of silylated hemiaminals with disulfides [246].

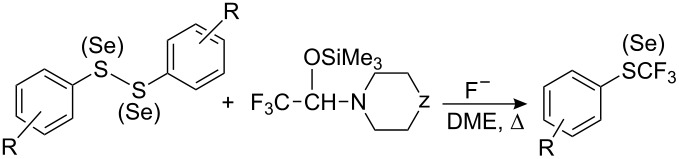					

R	S (Se)	Z	“F^−^”	*T*, °C	Yield, %

H	S	O	CsF	80	50
H	S	O	TBAT^a^	80	90
H	S	NCH_2_Ph	TBAT	60	78
H	S	NCH_2_Ph	TBAT	80	95
4-Cl	S	NCH_2_Ph	TBAT	80	95
H	Se	NCH_2_Ph	TBAT	80	92
4-Cl	Se	NCH_2_Ph	TBAT	80	75

^a^TBAT: Bu_4_N^+^ Ph_3_SiF_2_^−^.

The use of F^−^ anion as an alkaline agent (De-Shopge reagent, Bu_4_N^+^ Ph_3_SiF_2_^−^) in place of a strong base (*t*-BuOK) allows trifluoromethylation of aliphatic disulfides.

**4.3.5. “CF_3_^−^” anion from trifluoromethyl silanes**

Perfluoroalkyltrialkyl silanes in the presence of fluoride ion generate reactive R_F_ carbanions which have been used widely in synthesis [27–28,30–32,265]. For example, Ruppert’s reagent, CF_3_SiMe_3_ [266] and its tin analogue (CF_3_SnMe_3_) have been used for the nucleophilic introduction of a CF_3_ group to electrophilic sulfur for the preparation of trifluoromethyl sulfoxides and sulfones [267–269]. Trifluoromethyl trimethylsilane has also been used for the synthesis of aromatic trifluoromethyl sulfides and selenides (Table 17).

**Table 17:** Trifluoromethylation of sulfur and selenium compounds with Ruppert’s reagent.

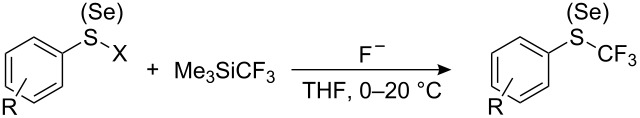				

X	F^−^	R	Yield, %	Ref.

Cl	TASF^a^	H	59	[270]
Cl	TASF	4-Cl	72	[270]
Cl	TASF	4-NO_2_	69	[270]
Cl	Bu_4_NF	4-NO_2_	14	[241]
SPh	Bu_4_NF	H	32 (43^b^)	[241]
CN	Bu_4_NF	H	70 (58^b^)	[271]
CN	Bu_4_NF	4-NO_2_	58	[271]
CN	Bu_4_NF	2,4-(OMe)_2_	30	[271]

^a^TASF = (Me_2_N)_3_S^+^ Me_3_SiF_2_^−^. ^b^ArSeCF_3_.

Reactions proceed easily in THF or light hydrocarbon solvents and the reaction can also be extended to aliphatic and heterocyclic [271] sulfur-trifluoromethylations. The data (Table 17) indicate that the source of the F^−^ anion exerts an important influence on the reaction of sulfenyl chlorides with CF_3_SiMe_3_ [267]. For example, in the presence of TASF *p*-nitrophenyl trifluoromethyl sulfide is formed in almost 70% yield, while the use of Bu_4_N^+^ F^−^ (even 2 equiv) under identical conditions gives only a 14% yield. In addition, in the reaction of diaryl disulfides with CF_3_SiMe_3_ it has been shown [241] that the best results are obtained when the Bu_4_NF is added with a syringe-pump rather than by ordinary dropwise addition.

Such trifluoromethylation reactions with CF_3_SiMe_3_ can also be catalysed with cyanide ion. However, this also results in competing side reactions where the cyanide attacks the disulfide directly and is especially problematic in the case of aliphatic disulfides [271].

**4.3.6. “CF_3_-anion” from ArSOCF_3_ and ArSO_2_CF_3_**

**Scheme 69:**
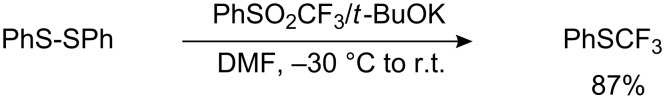
Trifluoromethylation of diphenyl disulfide with PhSO_2_CF_3_/*t*-BuOK.

Aryl trifluoromethyl sulfones react with CH_3_ONa to generate sodium arylsulfonates and fluoroform [272], and with Grignard reagents to generate aryl alkyl- or diaryl sulfones [273]. Also nucleophilic substitution of the pentafluoroethyl group can be induced in bis(pentafluoroethyl) sulfone by various nucleophiles [274]. Prakash et al. have adapted this chemistry for nucleophilic trifluoromethylation. Both phenyl trifluoromethyl sulfone or the corresponding sulfoxide on treatment with *t*-BuOK in DMF generate a CF_3_-adduct similar to that formed during fluoroform deprotonation [243,263], which is a useful trifluoromethylating agent for aldehydes, ketones and disulfides [248]. An example is shown in Scheme 69.

**Scheme 70:**
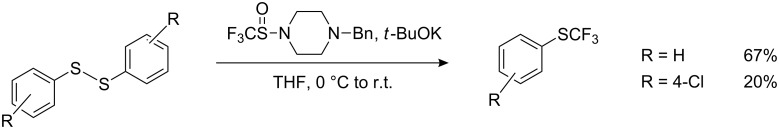
Amides of trifluoromethane sulfinic acid are sources of CF_3_^−^ anion.

On the other hand, under the same reaction conditions methyl trifluoromethyl sulfone does not function as a trifluoromethylating agent, whilst esters and amides of trifluoromethane sulfinic acid are good trifluoromethyl transfer agents [247] (Scheme 70).

However, trifluoromethylation strategies with aryl trifluoromethyl -sulfoxides, -sulfones, -sulfinates, and amides have to compete with cheaper reagents such as fluoroform, trifluoroacetic acid derivatives and trifluoromethyl halogenides. For the synthesis of aryl trifluoromethyl sulfides, it should be noted that these are prepared from sulfones, which are in turn synthesized from the same sulfides.

#### 4.4. Cationic perfluoroalkylation

Aryl perfluoroalkyl iodonium reagents as perfluoroalkylating agents were first developed by Yagupolski et al. [275]. Unlike perfluoroalkyl iodides, tolyl perfluoroalkyl iodonium chlorides react easily with sodium thiophenolates and selenophenolates at low temperature to form the corresponding aryl perfluoroalkyl sulfides and selenides as summarized in Table 18.

**Table 18:** Interaction of tolyl perfluoroalkyl iodonium chlorides with sodium thiophenolates and selenophenolate [275].

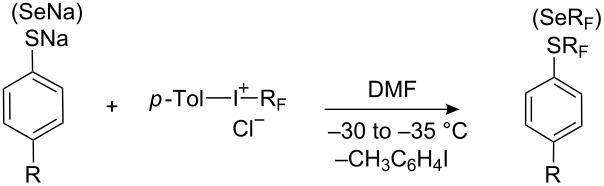			

R	R_F_	Yields, % (GLC) of	
		
		ArSR_F_	ArSeOR_F_^a^

H	C_3_F_7_	61 (81)	87
CH_3_	C_3_F_7_	71 (96)	–
NO_2_	C_3_F_7_	34 (56)	–
H	C_6_F_13_	41	45

^a^After chlorination and subsequent hydrolysis of corresponding selenides.

These iodonium salts even react with sodium *p*-nitrothiophenolate and while C_3_F_7_I does not react without some initiation [189] the C_3_F_7_ containing salts (Table 18) react readily. The yields of *p*-O_2_NC_6_H_4_SR_F_ (R_F_ = C_3_F_7_ and C_6_F_13_) are increased to a quantitative level by the use of iodonium tetrafluoroborate salts [276] instead of chlorides.

Similarly, perfluoroalkyl phenyl iodonium trifluoromethanesulfonates (FITS reagents) react with thiolates [277]: Perfluoroalkylation is selective for sulfur even in the presence of other functional groups (e.g. OH, NHMe, COOH, COOAlk). The preparation and application of R_F_ iodonium salts has been reviewed [33]. However, CF_3_ iodonium salts were not discussed, presumably due to their low stability.

**Scheme 71:**
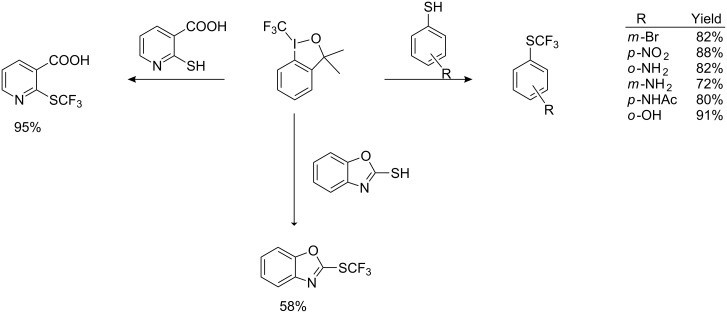
Trifluoromethylation of various thiols using “hyper-valent” iodine (III) reagent [279].

A “hyper-valent” iodine (III) compound containing a trifluoromethyl group, first synthesized in 2006 [278], appears to be quite stable. This moisture-sensitive reagent reacts with aromatic, heterocyclic and aliphatic thiols at low temperature (−78 °C) with the formation of the corresponding SCF_3_ derivatives in high yields (Scheme 71).

However, this attractive methodology has some drawbacks in that its synthesis involves four steps and trifluoromethylation products must be purified by chromatography to remove a side-product – 2-iodophenyl dimethyl carbinol.

**Scheme 72:**
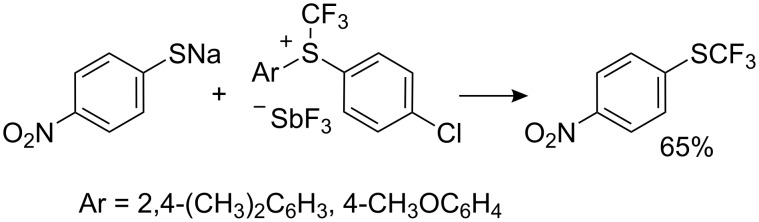
Trifluoromethylation of *p*-nitrothiophenolate with diaryl CF_3_ sulfonium salts [280].

Unlike iodonium salts, onium salts of the group VI elements appear to be more stable with CF_3_ group. Diaryl R_F_-sulfonium salts, where R_F_ = CF_3_, are readily synthesized from aryl trifluoromethyl sulfoxides [280]. Reaction of these reagents with sodium *p*-nitrothiophenolate affords the trifluoromethyl sulfide in good yield (Scheme 72).

**Scheme 73:**
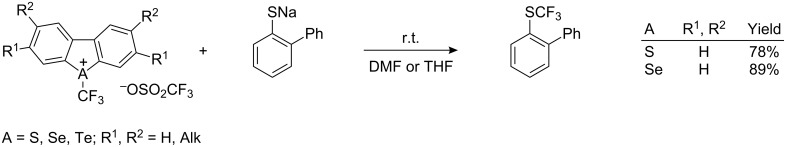
Trifluoromethyl transfer from dibenzo (CF_3_)S-, (CF_3_)Se- and (CF_3_)Te-phenium salts to thiolates [283].

It should be noted that for perfluoroalkylation is it necessary to use the diaryl sulfonium salts and not aryl alkyl sulfonium salts, since reaction of PhS^+^(CH_3_)CF_3_ BF_4_^−^, with *p*-nitrothiophenolate yields the SCH_3_ compound not the SCF_3_ derivative [280]. Subsequently, diaryl thiophenium, -selenophenium and -tellurophenium reagents have been developed with perfluoroalkyl groups attached to S, Se and Te [33,281–282] which can transfer perfluoroalkyl fragments to nucleophilic centers. In particular, the dibenzo (CF_3_)S-, (CF_3_)Se- and (CF_3_)Te-phenium systems have been investigated. For example, S(CF_3_)dibenzothiophenium triflate (A = S) reacts with sodium thiolate in DMF to give the *S*-trifluoromethyl derivative in high yield. The related selenophenium salt (A = Se) appears to be more effective in trifluoromethyl transfer (Scheme 73).

The same general reactivity is also observed in reactions of these reagents with aliphatic thiols. Dibenzoselenophenium triflate (A = Se, R^1^ and R^2^ = H) reacts much better with sodium dodecyl thiolate (yield of C_12_H_25_SCF_3_ is 87%) than the sulfur analogue (yield 47%) [283–284].

**Scheme 74:**
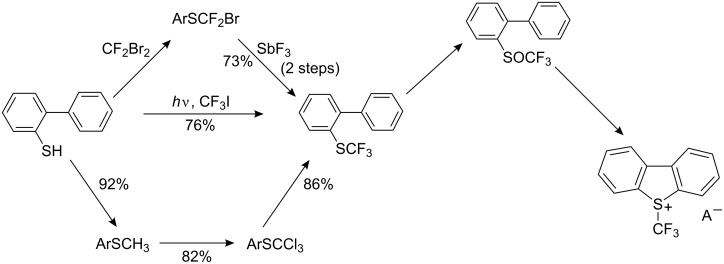
Multi-stage paths for synthesis of dibenzo-CF_3_-thiophenium salts [61].

On the whole R_F_ onium compounds are powerful perfluoroalkylating agents [33,281], however they are rather exotic reagents which require to be synthesized by multi-stage methods as illustrated in Scheme 74.

## Conclusion

A summary of the known methods for the synthesis of aromatic and heterocyclic perfluoroalkyl sulfides are presented. These involve perfluoroalkylation of thiols by single electron transfer, nucleophilic and electrophilic methods. The variety of methods reflects the level of interest chemists have given to generating this class of fluorine containing organic compounds. As a class of compounds, perfluoroalkyl sulfides find increasing utility in agrochemical and pharmaceutical applications.

A concise review concerning the preparation of selectively fluorinated ethers, thioethers, amines and phosphines was published [285] during preparation of this manuscript.
